# Analytic Linear Vibronic Coupling Method for First-Principles Spin-Dynamics Calculations in Single-Molecule Magnets

**DOI:** 10.1021/acs.jctc.2c00611

**Published:** 2022-10-21

**Authors:** Jakob K. Staab, Nicholas F. Chilton

**Affiliations:** Department of Chemistry, The University of Manchester, Manchester M13 9PL, U.K.

## Abstract

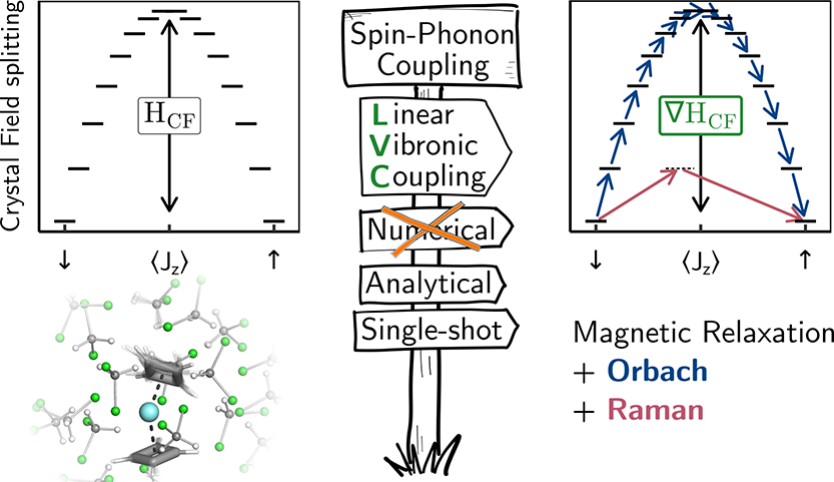

Accurate modeling of vibronically driven magnetic relaxation from ab initio calculations is of paramount importance to the design of next-generation single-molecule magnets (SMMs). Previous theoretical studies have been relying on numerical differentiation to obtain spin-phonon couplings in the form of derivatives of spin Hamiltonian parameters. In this work, we introduce a novel approach to obtain these derivatives fully analytically by combining the linear vibronic coupling (LVC) approach with analytic complete active space self-consistent field derivatives and nonadiabatic couplings computed at the equilibrium geometry with a single electronic structure calculation. We apply our analytic approach to the computation of Orbach and Raman relaxation rates for a bis-cyclobutadienyl Dy(III) sandwich complex in the frozen-solution phase, where the solution environment is modeled by electrostatic multipole expansions, and benchmark our findings against results obtained using conventional numerical derivatives and a fully electronic description of the whole system. We demonstrate that our LVC approach exhibits high accuracy over a wide range of coupling strengths and enables significant computational savings due to its analytic, “single-shot” nature. Evidently, this offers great potential for advancing the simulation of a wide range of vibronic coupling phenomena in magnetism and spectroscopy, ultimately aiding the design of high-performance SMMs. Considering different environmental representations, we find that a point charge model shows the best agreement with the reference calculation, including the full electronic environment, but note that the main source of discrepancies observed in the magnetic relaxation rates originates from the approximate equilibrium electronic structure computed using the electrostatic environment models rather than from the couplings.

## 1 Introduction

Single-molecule magnets (SMMs) have attracted much attention in recent decades due to their possible application in high-density data storage and spintronics devices.^1−3^ High-performance SMMs are molecules characterized by spin-dynamics that are slow enough to observe open magnetic hysteresis above ca. 40 K. In contrast to bulk magnetism which is retained through the formation of magnetic domains, SMMs do not rely on cooperative effects between a large number of electrons. Instead, hysteresis arises at the molecular level due to the presence of a doubly degenerate ground state with distinct orientations of the magnetic moment, that is, a center with large spin and orbital magnetic moments subject to large uniaxial magnetic anisotropy. On the one hand, the molecular nature of SMMs makes them amenable to chemical control and design, but on the other hand, they are highly sensitive to energy exchange with the environment giving rise to loss of magnetic memory.

An intrinsic energy barrier to reversal of the spin is one of the key ingredients of SMMs. Hence, much research has been focused on the enhancement of this barrier, which has been achieved by maximizing the magnetic anisotropy by designing suitable ligand fields. The record breaking dysprosocenium family,^4^ for instance, owes its huge magnetic anisotropy to negatively charged axial ligands which interact in a highly repulsive manner with the prolate 4f-electron density of the low-magnitude |*m*_*J*_| spin–orbit states.^5^ As a result, these states get lifted in energy leaving the *m*_*J*_ = ±15/2 states with oblate 4f-electron density as the ground doublet. This characteristic electronic structure leads to spin reversal via an Arrhenius-type magnetic mechanism at temperatures above ca. 60 K, known as the Orbach process, which exhibits rates with an inverse exponential dependence on temperature. However, a second, under-barrier mechanism called the Raman process, which follows a power-law temperature dependence, becomes active at lower temperatures.^6,7^

Both the spin reversal processes mentioned above are driven by the coupling between the electronic states and vibrations of the molecule itself as well as the phonons present in the surrounding environment. While Orbach relaxation (which arises from single-phonon absorption or emission) can be accurately described considering only optical phonons (i.e., intramolecular vibrations) for high-performance SMMs,^4,8^ the lower energy part of the vibrational spectrum composed of acoustic and pseudo-acoustic phonons must be included for a correct description of the two-phonon Raman process.^7,9,10^ In molecular crystals, (pseudo-) acoustic phonons arise from rigid body movements of molecules in the shallow intermolecular potential energy surface (PES). Hence, computing Raman rates without resorting to empirical parameterizations requires explicit account of the environment. This is a difficult task from a quantum chemical standpoint, and so previous computational work has mainly studied Orbach relaxation employing isolated gas-phase models of SMMs. Only recently, Briganti et al.^9^ presented the first complete study of the Orbach and Raman regimes employing an explicit atomistic condensed-phase environment.

State-of-the-art SMM spin dynamics simulations employ spin-phonon couplings derived from ab initio calculations in perturbative or open quantum system frameworks.^4,8,11^ In these calculations, the evaluation of spin-phonon coupling constants is the bottleneck for the overall computation. Most commonly, the ground multiplet of electronic states describing the low-temperature magnetism is projected onto a phenomenological spin Hamiltonian, for example, the crystal field potential  parametrized by a set of crystal field parameters (CFPs) {*B*_*q*_^*k*^} in a suitable basis of angular momentum operators, here total angular momentum ***Ĵ***.^12^ In the weak linear coupling regime, spin-phonon coupling encapsulated by first derivatives of the spin Hamiltonian parameters along the vibrational normal mode coordinates {*Q*_*j*_} is incorporated as a perturbation  following time-dependent perturbation theory (or more generally open quantum system theories) leading to the following partitioning of the Hamiltonian into zeroth order electronic  and vibrational  Hamiltonians and the spin-phonon interaction : .^10,13,14^ In the case of lanthanide complexes, the phenomenological crystal field Hamiltonian fully describes the splitting and mixing of the ground *J*-level, and CFPs are routinely determined based on multiconfigurational wave functions including spin–orbit coupling (SOC).^15^

Conventionally, CFP derivatives have been evaluated numerically by sampling explicit distortions of the complex along normal mode vectors around its equilibrium structure at the complete active space self-consistent field (CASSCF) level of theory.^4,9,10,14^ Approaches other than explicit electronic structure calculations exist, which may be cheaper, to evaluate spin Hamiltonian parameters at distorted geometries such as machine learning models.^16,17^ However, such methods ultimately depend on the sampling of ab initio parameters and the evaluation of numerical derivatives which suffer from various problems: (i) derivatives obtained from finite difference methods are known to intrinsically exhibit numerical and systematic errors governed by the distortion step size, and weak couplings arising from environmental degrees of freedom (DOFs) present a pathological case as they involve nuclear motion far away from the spin center; (ii) further numerical noise arises from the single-point CASSCF calculation followed by a crystal field projection which in itself is a numeric multistep procedure depending on convergence criteria; (iii) the practicalities of performing and maintaining tens of thousands of CASSCF calculations is laborious and error-prone as convergence and orbital rotations between the orbital spaces have to be monitored and scattered data complicates archiving efforts. These problems are in addition to the large computational burden required to perform many single-point distortion calculations at the CASSCF level. Therefore, an analytic approach for the computation of spin Hamiltonian parameter derivatives opens the door for more accurate and cheaper environment couplings.

The linear vibronic coupling (LVC) model is an attractive approach to analytically study a wide range of vibronic coupling phenomena due to its general and fundamental nature. Instead of computing the effect of nuclear dynamics on a preselected physical property, the LVC model takes a bottom-up approach by describing the unitary mixing of the electronic states at equilibrium geometry upon nuclear distortion.^18−23^ This effective change of basis can be applied to any equilibrium operator to yield physical properties at distorted geometries. The LVC model is based on a diabatic Hamiltonian parametrized by molecular gradients and nonadiabatic couplings (NACs). Analytic approaches to evaluate molecular gradients and NACs have been formulated for numerous levels of theory^24^ including multireference configuration interaction (MRCI) methods^25,26^ which were recently extended by density fitting techniques available in OpenMolcas.(27) Due to fact that the LVC model is based on parameters evaluated at a single geometry, it is also termed a “single-shot” approach, circumventing all of the problems featured by numerical differentiation schemes mentioned above.

In this work, we introduce a novel approach to obtain spin Hamiltonian parameter derivatives analytically from a single-shot LVC parametrization and use this new implementation to study the spin dynamics in a solvated bis-cyclobutadienyl Dy(III) sandwich complex , a proposed high-performance SMM,^8^ embedded in a frozen droplet of 22 dichloromethane (DCM) molecules. We have chosen this exemplar hypothetical molecule as it is the smallest chemically reasonable SMM related to the successful dysprosocenium family of sandwich compounds and minimizes the computational cost for our benchmarking purposes here. Likewise, we have chosen a single layer of DCM to represent a minimal amorphous frozen solution environment (DCM being an experimentally relevant choice^4^), which is crucial for obtaining the low-energy phonon density of states (DOS) that drives Raman relaxation.

Using this system, we assess the performance of our LVC derivatives and compare them to conventional derivatives obtained with numerical finite differences. The agreement is found to be within the typical errors of other approximations made in the calculations, and thus the simplicity and time savings make our LVC method hugely beneficial. We also benchmark different approximation schemes for representing the DCM environment (i.e., no environment, point charge, and point charge + dipole) in comparison to a reference calculation which includes the full DCM environment in the electronic Hamiltonian. Here, we find that a point charge representation exhibits the best compromise between computational cost and accuracy.

## 2 Theory

### 2.1 Numerical Differentiation

Here, we briefly outline numerical differentiation methods for comparison. Such methods are commonly employed to evaluate derivatives in cases where nothing but function values are available or the computation of analytic derivatives is too expensive. Its most fundamental flavor, the method of finite differences, is based on the approximation of the derivative by the limit of the differential quotient of a given mathematical function *f*(*x*) evaluated at inputs *x*_0_ – *h* and *x*_0_ + *h*.
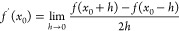
1In practice, the step size *h* is finite, and its magnitude controls the accuracy of the calculation in a trade-off between formula error at a large step size and truncation error at a small step size. The former, also known as secant error, dominates if *f* is not sufficiently linear in *x* on length scale *h* in which case, the secant slope described by the differential quotient does not serve as a good approximation of the true tangent derivative. The sources of the truncation error are numerical inaccuracies in the evaluation of differences in *f* at small step sizes. As differences get close to machine accuracy, floating point arithmetic exhibits the so called “catastrophic cancellation.” As a result, the careful choice of step size is crucial to the successful deployment of finite difference derivatives. Equation 1 corresponds to the method of central finite differences using a single step. More elaborate schemes employ more densely sampled grids which improve the formula error but increase the number of function evaluations linearly with the number of steps taken, such as the two-point central finite difference method employed in this work.^28^
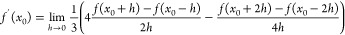
2In the case of the CFP derivatives ∂*B*_*k*_^*q*^/∂*Q*_*i*_, which are traditionally evaluated at discrete molecular distortions using single-point CASSCF calculations, the choice of step size runs into a dilemma. Set aside the fact that an appropriate step size is difficult to derive a priori, the fact that the function value {*B*_*k*_^*q*^} is vector-valued, that is, all ranks *k* and orders *q* are evaluated simultaneously, leads to a critical limitation. Without the presence of a “one-fits-all” choice, the optimal step size is inherently ill-defined, and the considerable computational cost of a CASSCF calculation renders individual step sizes an infeasible solution.

### 2.2 LVC Model

Within the Born–Oppenheimer (BO) approximation, the total Hamiltonian of a multielectron system can be expressed as , where *H*_MCH_ is the Molecular Coulomb Hamiltonian (MCH), the heart of electronic structure codes, which collects all electronic potential and kinetic energy terms and  is the SOC, Hamiltonian, accounting for the interaction between the electronic spin and the orbital angular momentum. The eigenbasis of  in terms of the MCH basis is obtained by diagonalization of ***H***, the matrix representation of the total Hamiltonian written in the MCH basis, that is, 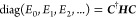
3yielding the energy eigenvalues of the total Hamiltonian *E*_0_, *E*_1_, *E*_2_, *...* along with the transformation .

Going beyond the BO approximation, vibronic coupling effects are incorporated in the diabatic picture by expanding the matrix representation of  written in the MCH states to first order in the nuclear displacements {*r*_*i*α_}, effectively coupling the PESs of the MCH states.^18,19^
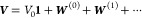
4
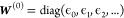
5
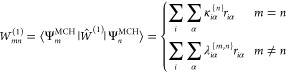
6Following the LVC model parametrization of Plasser et al.,^22^ the diabatic potential ***V*** is completely parametrized by the MCH equilibrium energies  as well as the intrastate and interstate vibronic coupling vectors  and . The summation indices *i* and α ∈ {*x*, *y*, *z*} run over the nuclear indices and directions in the Cartesian space. In our implementation, we intentionally omit the constant diagonal quadratic term *V*_0_ parametrized by the ground-state harmonic potential included by Plasser et al.^22^ as this term does not contain any state-specific vibronic coupling information but merely leads to an improved description of the PES for molecular dynamics applications.

The coupling vectors **κ**^{*n*}^ and **λ**^{*n*,*m*}^ are widely available from ab initio electronic structure calculations. **κ**^{*n*}^ is simply the nuclear energy derivative vector of the *n*th excited state, commonly employed in ground- or excited-state geometry optimizations, and **λ**^{*n*,*m*}^ is known in the literature as the NAC vector. At the MRCI level of theory, the NAC vector has been derived analytically and is contained in the so-called configuration interaction contribution to the derivative coupling vector.^26,27^

Evaluating eqs 4–6 at a small distortion and diagonalizing the resulting matrix ***V*** yield updated MCH energies in response to the distortion and the diabatic (MCH) to distorted transformation ***T***, that is, the mixing of the equilibrium MCH eigenstates upon distortion .
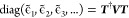
7Subsequently, ***T*** can be used to obtain the matrix representation of any equilibrium operator in the distorted MCH eigenbasis where the tilde denotes quantities endowed with this inherent geometry dependence, with .
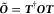
8Accordingly, the LVC model can be extended by additional, potentially geometry-dependent interactions included in the total Hamiltonian such as SOC. Within the atomic mean-field integral (AMFI) approximation,^29^ the matrix representation of the SOC interaction at distorted geometry  can be evaluated in a straightforward way since it is entirely parametrized by spin-free operators, that is, the AMFI matrix elements which are obtained at distorted geometry from eq 8. Subsequently, the SOC coefficients at distorted geometry  are obtained by diagonalization analogous to eq 3.

### 2.3 Spin Hamiltonians

Spin Hamiltonians are a common parametrization of typical interactions between angular momentum states observed in experimental and ab initio data. Common interactions between angular momenta include SOC and exchange coupling or couplings to external fields like the crystal field or Zeeman interaction. The applicability of spin Hamiltonians requires the electronic states of the system to be spanned by a tensor product basis of suitable angular momentum operators. For the sake of clarity, we focus our discussion on the theory of the ligand or crystal field splitting of lanthanide *J*-levels. However, we note that our methods are equally applicable to any other interaction terms linear in a set of coefficients scaling operators which act on angular momentum states.

The electronic spectrum of lanthanide complexes closely resembles the free ion terms due to the isolated nature of the spatially contracted 4f orbitals. The modification from the free ion is that each *J*-level is split by the effective crystal field imposed by the ligands. A common parametrization of the crystal field Hamiltonian is the operator equivalent method.^30^ This notation employs a linear combination of mutually orthogonal  operators of rank *k* and order *q* (0 ≤ *k* ≤ 2*J* and −*k* ≤ *q* ≤ +*k*) scaled by CFPs *B*_*k*_^*q*^ as well as constant operator equivalent factors θ_*k*_ tabulated for the ground multiplets of lanthanide ions. Even in the absence of molecular point symmetry, matrix elements of odd-*k* Stevens operators are zero between states arising from the same configuration (in this case 4f^*n*^) and can hence be omitted from the expansion.^31^
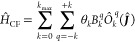
9The full Hamiltonian  written in a suitable angular momentum basis ***H***^angm^ and exhibiting matrix elements  can be employed to project out model parameters from ab initio calculations. The crystal field decomposition of ***H***^angm^ can be carried out by considering the projection properties of spherical tensor operators or simply by determining the least-squares minimizer  of eq 10.^12,15^
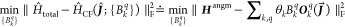
10To obtain the transformation from the eigenbasis of the total ab initio Hamiltonian to a given angular momentum basis, the appropriate basis operators, that is, squares and z-components of the ab initio spin (***S*^**), orbital (***L*^**), and total angular momentum  are evaluated in the MCH-SO eigenbasis defined by ***C*** introduced in eq 3 and simultaneously diagonalized via a step-wise block diagonalization approach yielding the basis transformation ***U*** and the diagonal matrices **β**^angm^ specifying the angular momentum quantum numbers.^32,33^
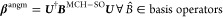
11These basis operators are chosen as they are a set of mutually commuting angular momentum operators, for example, , , , and  characterize the ^2*S*+1^*L*_*J*_-levels in lanthanide ions. From a formal standpoint of angular momentum addition, the set of basis operators is restricted by  and  generally leaving only the set , , , and  as an alternative choice. Subsequently, the arbitrary phases of the resulting angular momentum eigenstates are adjusted by requiring the *x*-component of their angular momenta to be purely positive and real, following the Condon-Shortley phase convention. Finally, according to the quantum numbers found during the block diagonalization, the ab initio angular momentum basis states are put into correspondence with the “ideal” model angular momentum states employed in the spin Hamiltonian which is built from the canonical angular momentum operators.

### 2.4 Analytic Spin Hamiltonian Parameter Derivatives

As all quantities required for the evaluation of spin Hamiltonian parameters can be obtained at distorted geometry, the LVC model could be in principle used as a computationally cheap generator of CFP values as a function of nuclear displacement entering a numerical differentiation scheme introduced in Section 2.1. However, here we go a step further and derive fully analytic derivatives of spin Hamiltonian parameters.

The nuclear derivative information enters the LVC model through the derivatives of the diabatic potential energy matrix elements.
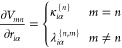
12As we will show in the following, the derivative information can be propagated analytically to all operators and eventually the total Hamiltonian matrix in the angular momentum basis required to project the CFP derivatives ∂*B*_*k*_^*q*^/∂*r*_*i*α_ = ∂_*i*α_*B*_*k*_^*q*^ based on the differentiated version of eq 10.
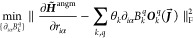
13To obtain the differentiated matrix elements of an operator in the MCH basis, eqs 8 and 7 are differentiated in the nuclear displacement.
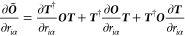
14
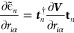
15
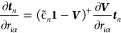
16where (...)^+^ denotes the Moore–Penrose inverse. Equations 15 and 16, which yield the derivatives of the eigenvalues  and corresponding eigenvectors {***t***_*n*_}, that is, columns of ***T***, are a result of eigenvalue perturbation theory on Hermitian matrices.^34,35^

Equations 14–16 are the workhorse of our implementation, facilitating the computation of spin and orbital angular momentum operator derivatives as well as derivatives of the total Hamiltonian in the MCH-SO basis. Equivalent techniques are used to propagate the derivative through the angular momentum basis transformation and yield the  derivatives to be employed in eq 13. Note that for the present application, the derivatives computed by eqs 13–16 are evaluated at the equilibrium geometry ***r*** = **0**. Hence, the final electronic basis in which the matrix element derivatives of  are evaluated corresponds to the angular momentum basis at equilibrium geometry.

To retain generality of the theory introduced in this work, we determine the derivatives in the Cartesian atomic coordinate basis. In the case of vibronic coupling applications, however, quantities need to be transformed into normal mode coordinates which correspond to a linear combination ***l*** of Cartesian displacements in which the harmonic ground-state potential described by the Hessian matrix ***H***_0_ is expressed as a sum of K ≤ 3*N* independent harmonic oscillators.
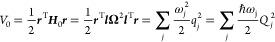
17The diagonal matrix Ω contains the frequencies {*ω*_*j*_} and is obtained through diagonalization of the mass-weighted Hessian
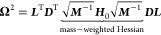
18where the matrix ***M*** contains elements *M*_*i*α,*i*α_ = *m*_*i*_ along its diagonal mass-scaling the corresponding atomic coordinates. The orthogonal 3*N* × *K* matrix ***D*** is employed to project out collective atomic displacements under which the potential energy is invariant, for example, rigid body translations or rotations of molecular systems, leading to the typical *K* = 3*N* – 6 (3*N* – 5) normal modes for nonlinear (linear) molecules. The columns of ***D***, representing a set of mass-weighted internal coordinates of the molecule, can be found by orthonormalizing all eigenvectors of the mass-weighted Hessian which correspond to non-zero eigenvalues against the six (five) coordinates corresponding to the mass-weighted infinitesimal translations and rotations via a Gram–Schmidt process.^36^ Finally, ***L*** is the orthogonal matrix which diagonalizes the mass-weighted Hessian in the internal coordinate frame ***D***. Upon comparison with eq 18, the linear transformation from 3*N* Cartesian displacements {*r*_*i*α_} to *K* normal mode coordinates {*q*_*j*_} is formed by . For convenience, our implementation works in dimensionless mass-frequency scaled normal coordinates , which defines the overall coordinate transformation.
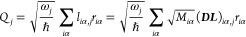
19
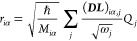
20The transformation of atomic derivatives to derivatives along normal mode coordinates follows from the chain rule.
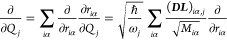
21

## 3 Computational Details

The spin dynamics calculations of DCM-solvated  were carried out following an established three-step protocol:^4,8,10,14,37^ (i) geometry optimization and harmonic frequency analysis at the density functional theory (DFT) level of theory, (ii) computation of electronic states and spin-phonon coupling at the CASSCF with SOC (CASSCF-SO) level, and finally (iii) solution of the semiclassical master equation to yield temperature-dependent magnetic relaxation rates.

The initial geometry of gas-phase  surrounded by 22 DCM molecules was generated using Packmol(38) with the spherical solvent ball option. All DFT calculations were carried out with Gaussian version 9 revision D.01^39^ using the pure PBE functional by Perdew et al.^40^ with Grimme’s D3 dispersion correction. Dysprosium was replaced by its diamagnetic analogue yttrium for which the Stuttgart RSC 1997 ECP basis was employed.^41^ Cyclobutadienyl (Cb) ring carbons directly coordinated to the central ion were equipped with Dunning’s correlation consistent triple-zeta polarized cc-pVTZ basis set and all remaining atoms with its double-zeta analogue cc-pVDZ.^42^ The whole system (including the solvent molecules) was geometry optimized until RMS (maximum) values in force and displacement corresponding to 0.00045 au (0.0003 au) and 0.0018 au (0.0012 au) were reached, respectively. After adjusting the isotopic mass of yttrium to that of dysprosium *m*_Dy_ = 162.5 u, vibrational normal modes and frequencies of the entire molecular aggregate were computed within the harmonic approximation. Electrostatic representations of the environment DCM molecules were evaluated for each isolated solvent molecule independently using the CHarges from ELectrostatic Potentials using a Grid-based method (ChelpG) scheme including only charges or charges and dipoles into the fit.^43^

The spin-phonon coupling of  was computed at the CASSCF level including scalar relativistic effects using the second-order Douglas-Kroll Hamiltonian and SOC through the AMFI approximation implemented in the restricted active space state interaction^29,44^ approach implemented in OpenMolcas version 21.06.^45^ The dysprosium atom was equipped with the ANO-RCC-VTZP, the ring carbons and directly coordinated solvent chlorine atoms with the ANO-RCC-VDZP, and the remaining atoms with the ANO-RCC-VDZ basis set.^46^ The resolution of the identity approximation with the acCD auxiliary basis was employed to handle the two-electron integrals.^47^ The active space of 9 electrons in 7 orbitals, spanned by 4f atomic orbitals, was employed in a state-average CASSCF calculation including the 18 lowest lying sextet roots which span the ^6^H and ^6^F atomic terms. Exclusion of the ^6^P states, as well as all quartets and doublets, is made based on the significant separation from the low-lying ^6^H and ^6^F terms. Inclusion of these states has negligible effect on the electronic structure of the ground ^6^H_15/2_ multiplet. Furthermore, including too many states can deteriorate the quality of the state-average molecular orbitals for the description of the relevant states. The solvent molecules were included using four different representations of increasing accuracy: neutral atoms (ghost; i.e., no contribution), ChelpG charges (charge-only), ChelpG charges and dipoles (charge + dipole), or a fully-electronic description employing the minimal ANO-RCC-MB basis set (MB). All CFP (derivatives) were calculated using our own implementations of the spin Hamiltonian parameter projection and the LVC approach described in Sections 2.2, 2.3, and 2.4 based on the state-average CASSCF density-fitting gradients and NACs involving all 18 sextet roots. For reference, numerical differentiation was carried out using a two-step central finite difference approach based on Cartesian atomic distortions by a step of 0.01 Å commonly employed in numerical differentiation schemes at the DFT level of theory.^39,48^

Spin dynamics calculations were carried out using the tau software, implementing a semiclassical approach used in previous work.^4,8,11,49^ Transition rates between different states are obtained by integrating the spin-one-phonon and spin-two-phonon rate expressions^50^ over the phonon DOS, weighted by Bose–Einstein occupation factors. The spin-phonon matrix elements are evaluated in the equilibrium CFP eigenbasis using CFP derivatives in normal mode coordinates. The vibrational DOS is constructed from uncalibrated harmonic frequencies broadened by antilorentzian line shapes of a constant line width parameter FWHM = 10 cm^–1^.^8^ Further details, including rate expression for the Orbach and Raman regimes, are given in Section S1.^8,50^

## 4 Results and Discussion

**Figure 1:**
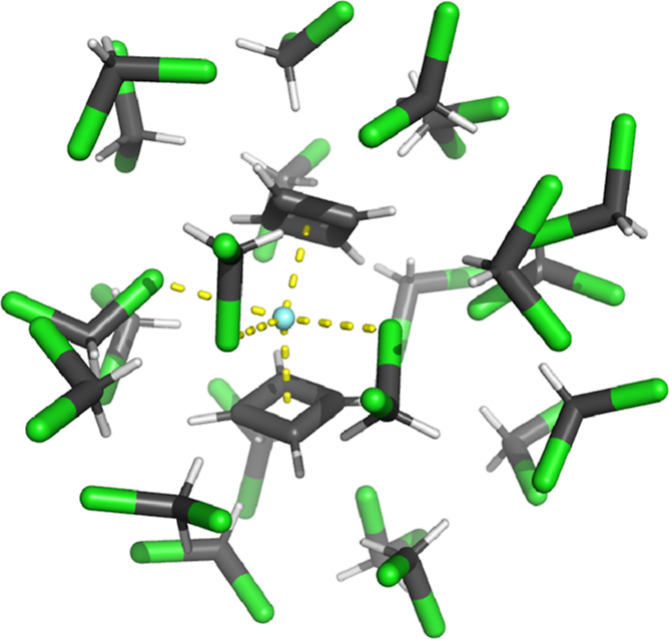
Geometry of the optimized  structure in frozen DCM solution. Hydrogen atoms are shown in white, carbon in gray, chlorine in green, and the central dysprosium atom in light blue. Coordinating bonds from the Cb ligands and the three coordinating DCM molecules are depicted with yellow dashed lines.

Herein, we consider the proposed  SMM. However, due to the small size of the Cb ligands, the molecule has a undersaturated coordination sphere, and polar solvents like DCM would be expected to coordinate to the Dy ion. Indeed, our computational solvation and geometry optimization results show that three DCM molecules coordinate directly via their chlorine atoms and disrupt the axial arrangement of the Cb ligands. Consequently, we subsume these three DCM molecules into the definition of the SMM. The final geometry of the system is shown in Figure 1.

### 4.1 Benchmark of Analytic LVC Derivatives

Atomic CFP derivatives were evaluated for all nuclear SMM () and environment (19 × DCM) DOFs using the LVC method as well as traditional central finite differences from data computed at explicitly distorted geometries. The Pearson correlation coefficient ρ is used to quantitatively assess agreement between derivatives obtained using either method, which is computed independently for derivatives associated with the environment ρ_env_ and the SMM DOFs ρ_SMM_, respectively.

While the small size of the model system suggests that a fully electronic description of the environment (i.e., minimum basis (MB) set on all DCM molecules) would be possible, we ran into memory constraints during calculation of the gradients and NACs on our computer hardware. Thus, we turned to an electrostatic model of the environment (i.e., ChelpG-derived charges) to compare the derivative methodologies; this choice is validated further in Section 4.2.

**Figure 2:**
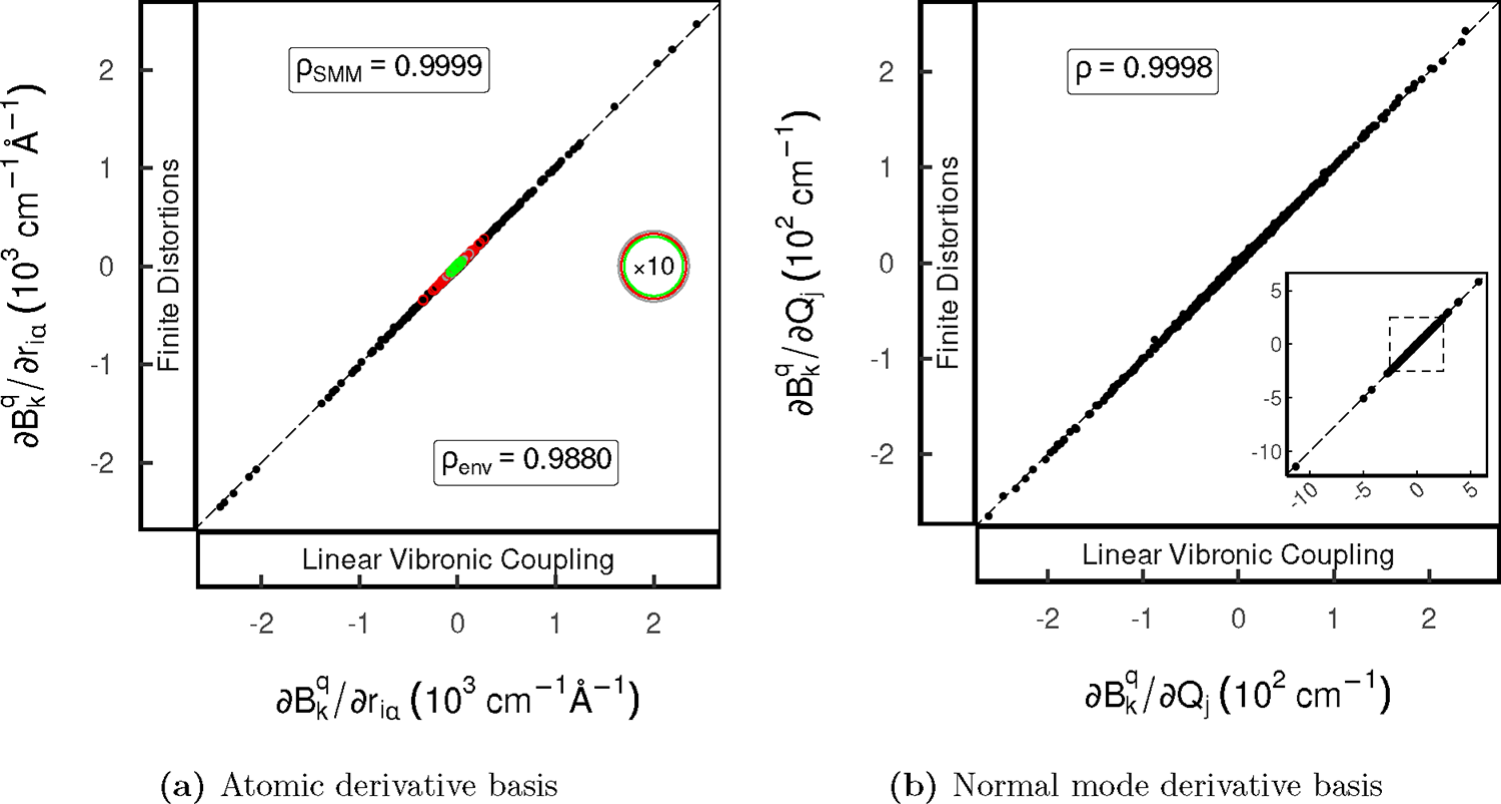
Correlation scatter plots of the atomic (a) and normal mode (b) CFP derivatives, comparing the reference finite difference data to values computed with the analytic LVC approach, modeling the environment by point charges. Environmental and SMM (and normal mode) DOFs are depicted with unfilled and solid dots, respectively. The values of atomic CFP derivatives associated with environmental DOFs colored in gray (DCM-carbon), red (DCM-hydrogen), and green (DCM-chlorine) are scaled by a factor of 10 for better visibility.

Considering first the atomic Cartesian displacements (Figure 2a) across environment and SMM DOFs, the datasets show excellent agreement with the correlation coefficients of ρ_SMM_ = 0.9999 and ρ_env_ = 0.9880. The poorer correlation for the environmental DOFs owes to the weak influence of these motions on the electronic structure of the SMM, which leads to small magnitude changes in the CFP compared to those for the SMM DOFs (see Section S2 for explicit Δ*B*_*q*_^*k*^ = *B*_*q*_^*k*^(***r***) – *B*_*q*_^*k*^(0) scans). In the case of the environment DOFs, significant numerical noise arises due to premature convergence of the underlying CASSCF single-point calculations. Even though this could be mitigated by tighter convergence criteria, convergence behavior can be difficult to predict and is system-dependent; in this sense, our LVC method has a distinct advantage. These scans also show that the finite step size of 0.01 Å is appropriate in the case of the SMM DOFs as the scans show a high degree of linearity and negligible numerical noise demonstrating a low secant and truncation error, respectively. At close inspection of the correlation plot, the magnitude of the derivatives obtained by the analytic LVC method systematically exceeds the data obtained using finite distortions slightly, where a linear fit yields slope *m* = 1.0064(2).

Calculation of finite difference derivatives in the atomic Cartesian basis is especially sensitive to numerical errors (as coupling strengths vary for different DOFs by orders of magnitude), and examining the derivatives in the normal mode basis should alleviate this issue somewhat. The overall correlation (which can no longer be separated into environment and SMM DOFs) in this case is ρ = 0.9998, indicating that the discrepancies in the weakly coupled environmental DOFs are washed out in the normal mode basis [Figure 2b; we note that the systematic deviation between the two methods is retained in the normal mode basis with *m* = 1.0082(2)]. However, still there is a large range of coupling strengths across the modes, leading to a large dispersion in the relative deviation of the calculated couplings (Figure S7).

Neither analytical nor numerical CFP derivatives can serve as the ”gold standard” benchmark as the computation of both involve a different set of approximations and sources of errors (mainly truncation of the diabatic basis in the case of the LVC method and numerical inaccuracies in the case of finite difference derivatives), rendering the quantitative assessment of their respective accuracy challenging. However, we can further compare the accuracy of the CFP derivatives by examining the translational invariance condition, defined in eq 22, for both methods. Just as the molecular gradients and NACs are invariant under translation, so should be the CFP derivatives within each rank (*k*) and order (*q*). The right-hand part of eq 22 shows that the sum of all ∂*B*_*q*_^*k*^/∂*r*_*i*α_ for a given rank, order, and Cartesian direction should be zero. Taking the absolute value of these sums and representing it as a percentage of the equilibrium *B*_*q*_^*k*^ value, Figure S8 shows that the invariance condition is far more strongly obeyed by the LVC-based analytic CFP derivatives than by the finite difference-based derivatives by around 6–8 orders of magnitude. This is likely due to errors stemming from the numerical differentiation (including premature CASSCF convergence) and/or from the fact that the calculation of CFPs, in particular the orbital angular momentum integrals, is not gauge invariant.
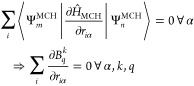
22

Our final comparison is on computational cost. In the case of our benchmark system, we observe a five-fold reduction in computational time when using the LVC approach compared to a two-point central finite difference scheme. As the computational performance of both methods are highly dependent on the molecular system and the computational setup, it is more instructive to consider their computational scaling in practice as illustrated in Figure S9. The evaluation of conventional finite difference CFP derivatives requires the computation of atomic integrals, CASSCF wave function, and SOC at 2 × 3 × *N*_atoms_ × *N*_steps_ geometries. The LVC model on the other hand is parametrized by  gradients. The overall computational cost of the gradient evaluation is effectively independent of the number of point multipoles in the environment. This is due to the fact that the electrostatic potential imposed by the environment enters the MCH through one-electron operators. Instead, the main cost of the gradient evaluation is the differentiation of the electron–electron repulsion potential. Hence, we predict our LVC-based method to be vastly more efficient in cases where many environmental DOFs are required, such as in true crystalline solids.

### 4.2 Effect of the Environment Model on the CFP Derivatives

**Figure 3:**
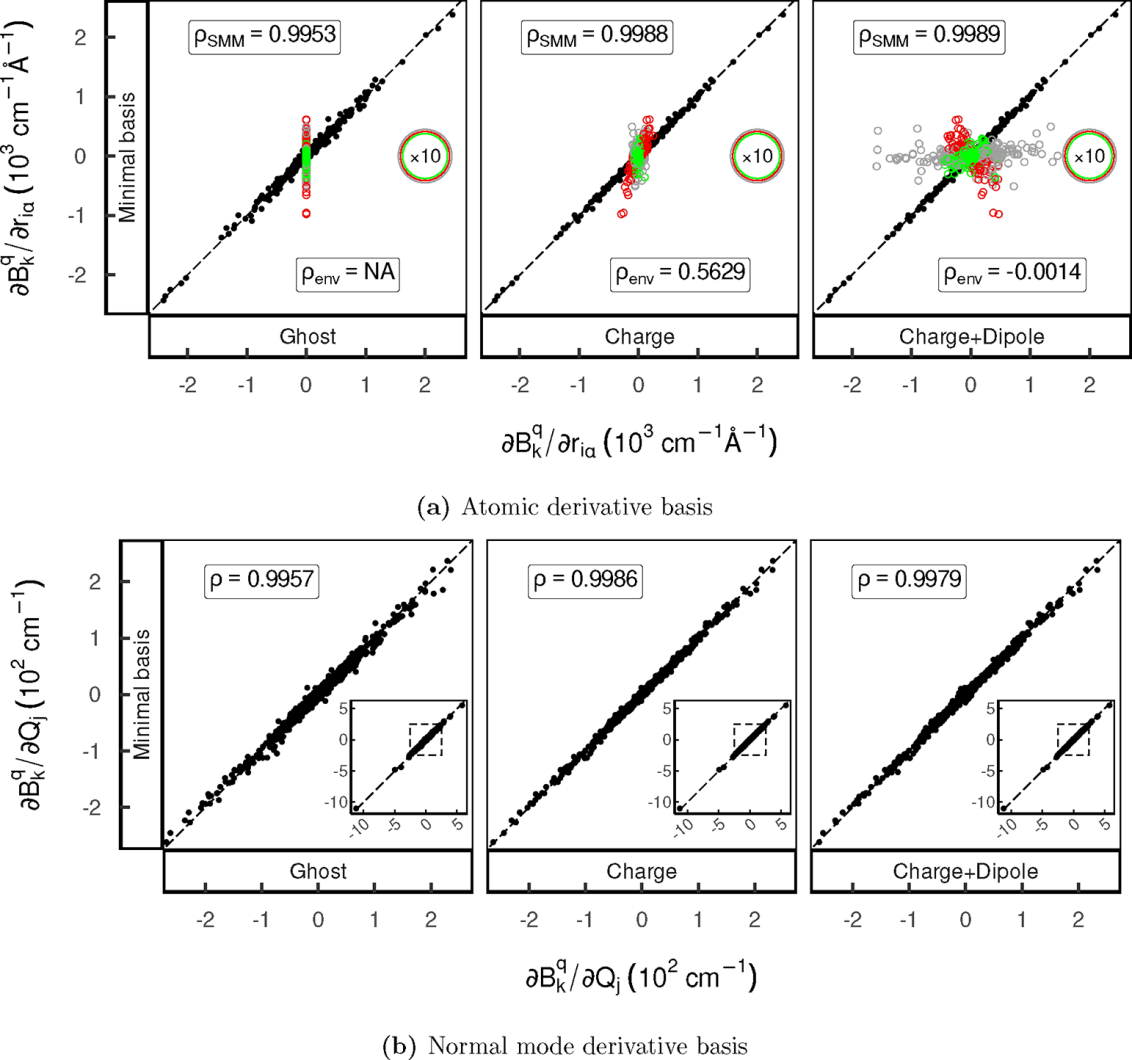
Correlation scatter plots of the atomic (a) and normal mode (b) CFP derivatives, comparing the reference MB data to the different environment models. Environmental and SMM (and normal mode) DOFs are depicted with unfilled and solid dots, respectively. The values of atomic CFP derivatives associated with environmental DOFs colored in gray (DCM-carbon), red (DCM-hydrogen), and green (DCM-chlorine) are scaled by a factor of 10 for better visibility.

In this section, we investigate the sensitivity of the calculated spin-phonon couplings to different environment models. To this end, CFP derivatives calculated under different electrostatic multipole representations of the environment are compared to the explicit MB representation by means of the Pearson correlation coefficient and correlation scatter plots (Figure 3), giving insight into their relative accuracy. Mutual correlation plots of all environmental representations are shown in Figures S10 and S11.

First, focusing on the SMM DOFs (defined in the Cartesian atomic displacement basis) shown in Figure 3a, charge-only and charge + dipole representations slightly outperform the ghost (i.e., no) environment. Thus, the coupling of the SMM DOFs is only weakly sensitive to details of the environment. Even though the charge + dipole representation should improve the description of the electrostatic potential of the environment, it gives a negligible improvement in the derivatives.

Second, considering the coupling to environment DOFs, a more pronounced dependence on model choice is observed. As the environmental DOFs do not interact with the electronic states of the SMM in the ghost representation, their coupling is zero by definition. Between the multipole expansion representations of the environment, the charge-only model surprisingly correlates significantly better with the MB representation than the charge + dipole model (ρ_env_ = 0.5629 cf. ρ_env_ = −0.0014). The reason for this result is that the environmental charges and dipoles are kept static and with fixed orientation throughout the distortion (i.e., they are not perturbed by the molecular distortion). Even though the charge + dipole model more accurately describes the electrostatic potential at equilibrium, it breaks down upon geometric distortion of the environment. Atomic charges are much less constrained when dipoles are included in the ChelpG fit, and so they lose their “chemical intuition.” On the other hand, when only charges are fitted, they are “chemically sensible” and yield an electrostatic potential which is meaningful over a wider range of molecular conformations. This is clear from inspection of the charges and dipoles obtained from the ChelpG analysis (Table 1). The charges of DCM carbon and hydrogen atoms are negative and positive when only charges are fitted, as expected based on differences in electronegativity; however, the charges change sign upon introduction of dipoles. In the normal mode basis, the quality of the derivatives for different environment representations follows the same ranking as in the Cartesian atomic basis, albeit that deviations to the reference calculation partially average out during the transformation (Figure 3b).

**Table 1:** Atomic Point Charges and Dipoles Using a Charge-Only and Charge + Dipole ChelpG Fit of the Molecular Electrostatic Potential^a^

expansion	atom	charge	dipole magnitude
charge	H	0.168 ± 0.004	
charge	C	–0.17 ± 0.01	
charge	Cl	–0.084 ± 0.003	
charge + dipole	H	–0.47 ± 0.02	0.43 ± 0.01
charge + dipole	C	2.16 ± 0.03	0.11 ± 0.02
charge + dipole	Cl	–0.608 ± 0.006	0.789 ± 0.008

a The uncertainties denote the standard deviation among all environment DCM molecules.

### 4.3 Magnetic Relaxation Rates

**Figure 4:**
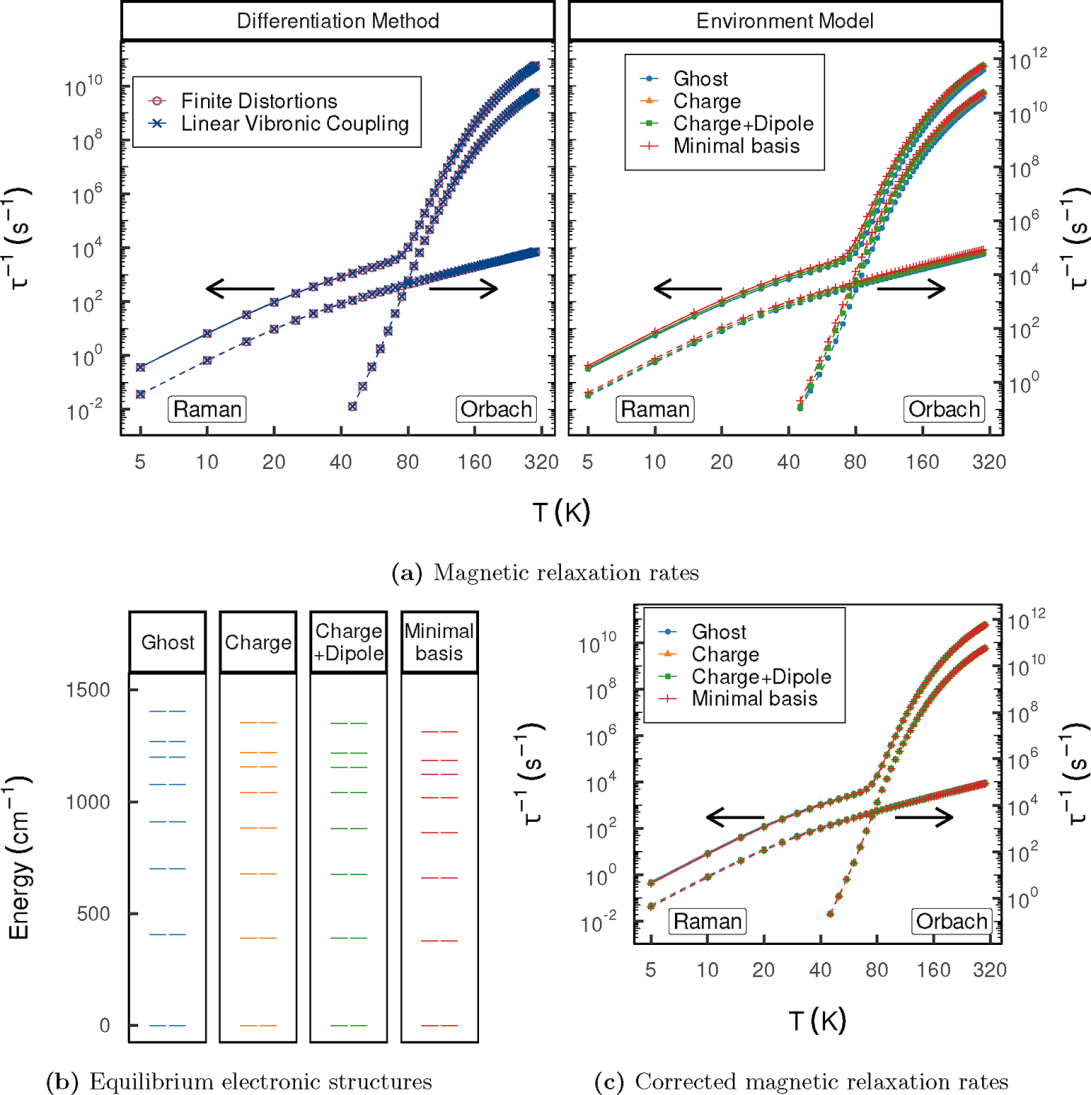
(a) Magnetic relaxation profiles of  employing different differentiation schemes (left) and environment models (right). The total rates indicated by the solid lines are plotted on the primary (left) axis, and the rates of the individual processes represented by the dashed line are plotted on the secondary (right) axis. The axes are shifted relative to each other by a factor of ×10 for improved visualization. (b) Equilibrium CASSCF-SO electronic energy spectrum of the ^6^H_15/2_ level employing different environment models. (c) Corrected magnetic relaxation profiles computed by combining the spin-phonon couplings of the electrostatic environments with the equilibrium electronic structure of the reference MB calculation.

While we have shown that the LVC method produces derivatives of equal and likely higher quality than those obtained with finite differences, we now seek to examine the impact on the prediction of temperature-dependent spin reversal rates. We choose this observable as it characterizes the performance of a SMM and hence is the most important metric in contemporary SMM research. At low temperatures, the calculated rates show the characteristic Raman regime (Figure 4a), approximately linear on a log–log scale, and above 80 K, the Orbach process takes over. We observe almost no deviation between the results computed from numerical derivatives and LVC derivatives. Thus, we are confident that our implementation is robust and that it will prove to be a powerful technique to obtain analytic vibronic coupling parameters for a wide range of cases in spectroscopy and magnetism.

The dependence on the environment model is also very weak, leading to maximum variations of a factor of ∼2 and ∼4 in the case of the charge-only or charge + dipole and ghost representations, respectively, which reflects the relatively weak solvent dependence also observed experimentally in other SMMs.^4^ Over the whole temperature range, the ranking of the accuracy of environmental representation follows the same ordering as observed for the derivatives: charge-only ≈ charge + dipole > ghost; the electrostatic environment models systematically underestimate the reference rates.

The major source of the discrepancies observed in comparison of the rates for different environmental representations, however, is not inaccuracies introduced through the spin-phonon couplings but the equilibrium eigenstates computed with the different environment approximations. The ^6^H_15/2_ ground-level energy spectrum shown in Figure 4b reveals that employing the ghost environment leads to an overestimation of the crystal field splitting which is partially alleviated at thr charge-only and charge + dipole environment. This reflects the deficient description of the perturbation of the electronic states by the environment due to the approximate nature of the electrostatic multipole expansion and neglect of polarization effects as well as nonclassical interactions. These changes affect the rates because the electronic energies happen to be close to peaks in the vibrational DOS (Figure S12).

By combining the equilibrium electronic structure from the reference MB calculation (i.e., using the MB-calculated equilibrium CFPs to define the eigenbasis) while using the spin-phonon couplings from various environmental representations in the rate calculations (see Section S1), the resulting rates are almost completely recovered compared to the reference MB rates (Figure 4c). Hence, we propose a method where low-level couplings are combined with a high-level electronic structure. Since the couplings are evaluated based on the more demanding gradient calculations while the equilibrium electronic structure is computed in a single-point calculation, this enables a critical reduction in the computational cost.

## 5 Conclusions

A novel analytic approach, combining the LVC model with analytic CASSCF gradients and NACs, for the evaluation of LVCs in SMMs was introduced and subsequently applied to calculate Orbach and Raman relaxation rates in a proposed bis-cyclobutadienyl Dy(III) SMM^8^ solvated in a monolayer of DCM molecules, reproducing relaxation rates obtained via conventional finite difference differentiation to high fidelity.

Not only is our method transferable to other levels of theory, which makes it a highly versatile tool, the LVC approach can also be systematically improved and extended to higher-order couplings by, respectively, including higher excited state or higher-order vibronic coupling parameters.^51^ This opens doors for the highly anticipated augmentation of the current CASSCF treatment of spin-phonon coupling with dynamic correlation via multireference perturbation theory such as complete active space second-order perturbation theory (CASPT2),^52−54^ which has not been amenable to numerical differentiation schemes due to the presence of PES discontinuities.

We have further demonstrated that the vibronic coupling to low-energy rigid body vibrations involving molecules in the environment, crucial to the modeling of the Raman process, can be included using atomic point charges outperforming higher multipole expansions. We note, however, that the main sources of discrepancies observed in the relaxation rates are not introduced through the approximative vibronic coupling of point charges. Instead, we have found that the sensitivity of the equilibrium electronic structure to the electrostatic model environments is mostly responsible for the environment dependence of spin-dynamics.

We expect that the LVC approach for the evaluation of spin-phonon couplings in SMMs will be greatly valuable for future spin-dynamics calculations as the field moves on from gas phase models to more realistic solid-state environments. Our method enables high numerical accuracy and major computational savings as weak vibronic couplings of extended atomistic environment models are included analytically at effectively no extra cost.

## Supporting Information Available

Brief review of the spin dynamics theory used to compute magnetic relaxation including expressions for Orbach and Raman rates; comparison between CFP scans obtained from explicit single-point CASSCF calculations and the LVC method; comparison of the finite difference and LVC-derived CFP derivatives based on relative deviation as well as translational invariance; comparison of the computational cost of spin-phonon coupling calculations using finite difference differentiation versus the LVC method; correlation scatter plots of spin-phonon coupling data employing different electrostatic environment models; and overlap between vibrational DOS and electronic states of the ground ^6^H_15/2_ multiplet under different environment models (PDF)

## Notes

The analytic LVC-based differentiation algorithm is implemented as part of our in-house python packages spin-phonon_suite and angmom_suite freely available from the PyPI repository. The research data for this work are available at https://doi.org/10.48420/21076921.
