# Correction to Analytic Linear Vibronic Coupling Method for First-Principles Spin-Dynamics Calculations in Single-Molecule Magnets

**DOI:** 10.1021/acs.jctc.4c00239

**Published:** 2024-03-29

**Authors:** Jakob Staab, Nicholas Chilton

The implementation of the coordinate transformation from atomic to dimensionless mass-frequency scaled normal mode coordinates expressed in eqs 19 and 20 of our paper^1^ was incorrect. It involved a spurious factor of , where μ_*j*_ is the reduced mass of normal mode *j*. This gave spin-phonon couplings, expressed as derivatives of the Crystal Field Parameter (CFP) parameters along the normal mode coordinates {*∂B*_*k*_^*q*^/*Q*_*j*_}, which were overestimated by a factor of ∼1 to 5. While this error does not impact any of our conclusions, as correlations between couplings calculated using different differentiation methods or environmental representations are relative, we take the opportunity to present the correct data to ensure our work is reproducible and a true representation of the relaxation dynamics of the hypothetical [Dy(Cb)_2_(DCM)_3_]^−^ compound. In fact, due to the nature of our study, none of the numbers quoted in the original paper^1^ have changed, and it is only the figures that need correction. Here, we reproduce the figures that need corrections: Figures 1, 2, 3, 4 and 5, corresponding to Figures 2b, 3b, S11, 4a, and 4c in ref (1).

To solve the error, we scaled the erroneous couplings {*∂B*_*k*_^*q*^/*Q*_*j*_} by mode-specific scaling factors  and recomputed the temperature dependent magnetic relaxation rates.

We further used updated expressions to evaluate the Raman rates replacing eqs 3–6 in the SI of ref (1) with the expressions given in eqs 46–49 of ref (2). Note that these expressions need to be amended by a factor of 1/4 for compatibility with the normal mode coordinate definition used in the present work to which we issue the correction (see eq 19), which is different from the normal mode coordinates we have used more recently in refs (2−4).

**Figure 1:**
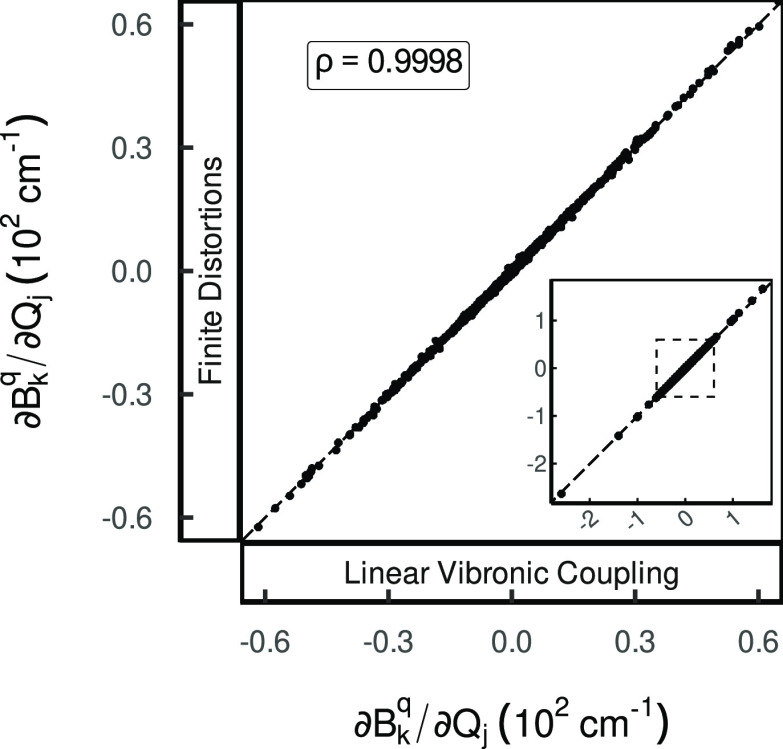
Correlation scatter plots of the normal mode CFP derivatives, comparing the reference finite difference data to values computed with the analytic Linear Vibronic Coupling (LVC) approach, modeling the environment by point charges; formerly Figure 2b in ref (1).

**Figure 2:**
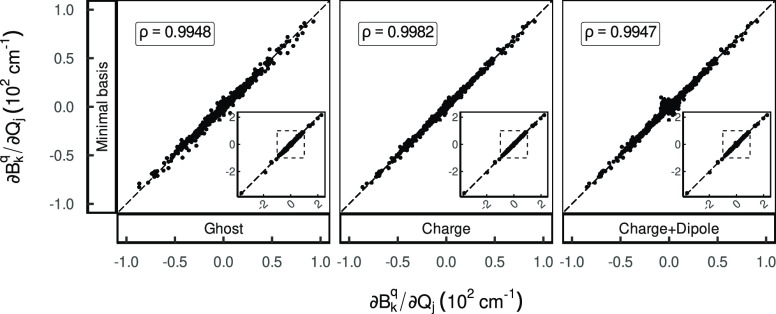
Correlation scatter plots of the normal mode CFP derivatives, comparing the reference Minimum Basis (MB) data to the different environment models; formerly Figure 2b in ref (1).

**Figure 3:**
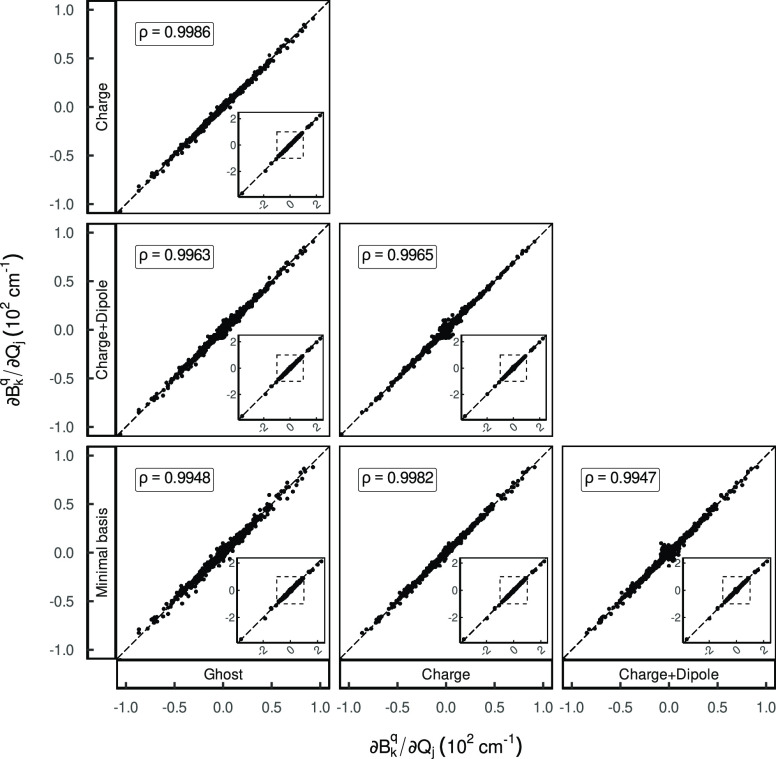
Mutual correlation scatter plots of the CFP derivatives in the normal mode basis between different environment representations; formerly Figure S11 in ref (1).

**Figure 4:**
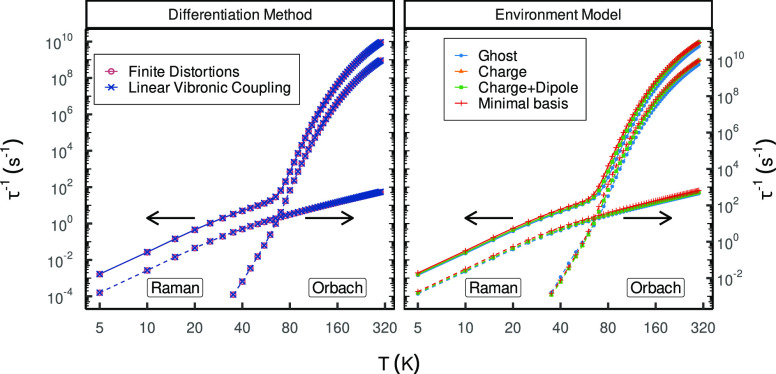
Magnetic relaxation profiles of [Dy(Cb)_2_(DCM)_3_]^−^ employing different differentiation schemes (left) and environment models (right). The total rates indicated by the solid lines are plotted on the primary (left) axis, and the rates of the individual processes represented by the dashed line are plotted on the secondary (right) axis. The axes are shifted relative to each other by a factor of ×10 for improved visualization; formerly Figure 4a in ref (1).

**Figure 5:**
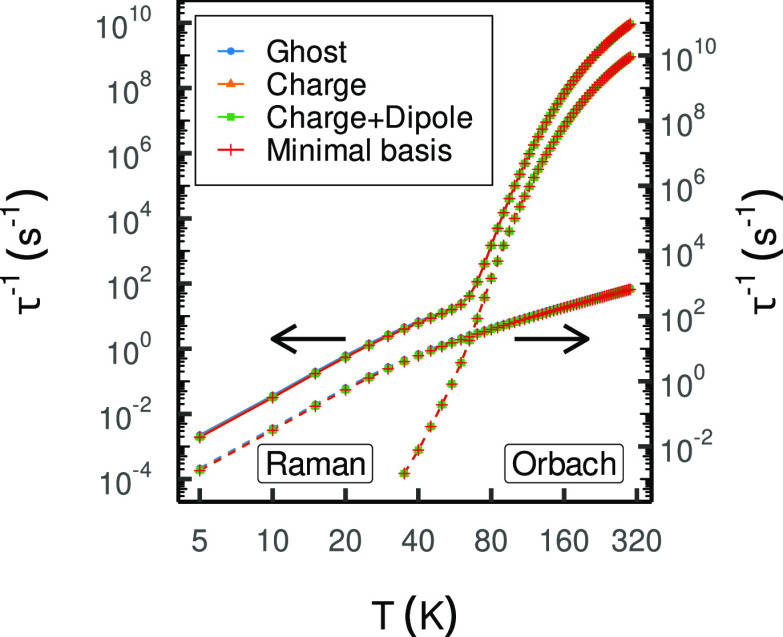
Corrected magnetic relaxation profiles computed by combining the spin-phonon couplings of the electrostatic environments with the equilibrium electronic structure of the reference MB calculation; formerly Figure 4c in ref (1).
