# Supplementary material for: Analytic Linear Vibronic Coupling Method for First-Principles Spin-Dynamics Calculations in Single-Molecule Magnets
Source: J Chem Theory Comput. 2022 Oct 21;18(11):6588–99. doi: 10.1021/acs.jctc.2c00611 (PMC9648194; doi:10.1021/acs.jctc.2c00611)
Supplement: Supplementary file 1 — ct2c00611_si_001.pdf [file ct2c00611_si_001.pdf]

Supplementary Information

An analytic linear vibronic coupling method for first-principles spin-dynamics calculations in single-molecule magnets

Jakob Staab*[1] and Nicholas Chilton*[1]

[1]Department of Chemistry, The University of Manchester, Manchester M13 9PL, U.K.

October 14th, 2022

Contents

S1 Spin dynamics 2

S2 Displacement scans 4

S3 Relative deviation of the differentiation methods 10

S4 Translational invariance of numerical and analytic derivatives 11

S5 Computational benchmark of derivation method 12

S6 Full correlation matrix of CFP derivatives 13

S7 Vibrational density of states 15

*email: jakob.staab@postgrad.manchester.ac.uk, nicholas.chilton@manchester.ac.uk

S1 Spin dynamics

To obtain temperature dependent magnetic relaxation rates, we use theory outlined in previous works,[1,2] which we extended with Raman expressions adapted from Lunghi's excellent work.[3] The relaxation rates γ_{fi} between the electronic eigenstates i and f define the semi-classical master equation, and are calculated independently for the Orbach (equation 1 and 2) and Raman (equation 3 to 6) mechanisms. The electronic eigenbasis is determined by diagonalising the equilibrium crystal field Hamiltonian, yielding the eigenvalues $\{E_i\}$ and the transformation from the angular momentum basis to the eigenbasis. The crystal field parameter derivatives in normal mode coordinates are then evaluated in the equilibrium electronic eigenbasis, defining the matrix elements $\langle f|\hat{V}_j|i\rangle = \langle f|\sum_{k=2}^{6}\sum_{q=-k}^{+k}\theta_k \frac{\partial B_k^q}{\partial Q_j}\hat{O}_k^q(\hat{\boldsymbol{J}})|i\rangle$ that enter the rates below.

$$\gamma_{fi}^{-} = \frac{2\pi}{\hbar}\sum_j \int \left|\langle f|\hat{V}_j|i\rangle\right|^2 \frac{\bar{n}_j}{2}\ \delta(E_f - E_i - \hbar\omega_j)\ \rho_j(\hbar\omega_j)\ \mathrm{d}\hbar\omega_j \tag{1}$$

$$\gamma_{fi}^{+} = \frac{2\pi}{\hbar}\sum_j \int \left|\langle f|\hat{V}_j|i\rangle\right|^2 \frac{\bar{n}_j + 1}{2}\ \delta(E_f - E_i + \hbar\omega_j)\ \rho_j(\hbar\omega_j)\ \mathrm{d}\hbar\omega_j \tag{2}$$

$$\gamma_{fi}^{--} = \frac{2\pi}{\hbar}\sum_{j\geq k}\left(1 - \frac{3}{4}\delta_{jk}\right)\iint \left|\sum_c \frac{\langle f|\hat{V}_j|c\rangle\,\langle c|\hat{V}_k|i\rangle}{E_c - E_i - \hbar\omega_k} + \frac{\langle f|\hat{V}_k|c\rangle\,\langle c|\hat{V}_j|i\rangle}{E_c - E_i - \hbar\omega_j}\right|^2$$
$$\frac{\bar{n}_j}{2}\frac{\bar{n}_k}{2}\ \delta(E_f - E_i - \hbar\omega_j - \hbar\omega_k)\ \rho_j(\hbar\omega_j)\rho_k(\hbar\omega_k)\,\mathrm{d}\hbar\omega_j\,\mathrm{d}\hbar\omega_k \tag{3}$$

$$\gamma_{fi}^{++} = \frac{2\pi}{\hbar}\sum_{j\geq k}\left(1 - \frac{3}{4}\delta_{jk}\right)\iint \left|\sum_c \frac{\langle f|\hat{V}_j|c\rangle\,\langle c|\hat{V}_k|i\rangle}{E_c - E_i + \hbar\omega_k} + \frac{\langle f|\hat{V}_k|c\rangle\,\langle c|\hat{V}_j|i\rangle}{E_c - E_i + \hbar\omega_j}\right|^2$$
$$\frac{(\bar{n}_j + 1)}{2}\frac{(\bar{n}_k + 1)}{2}\ \delta(E_f - E_i + \hbar\omega_j + \hbar\omega_k)\ \rho_j(\hbar\omega_j)\rho_k(\hbar\omega_k)\,\mathrm{d}\hbar\omega_j\,\mathrm{d}\hbar\omega_k \tag{4}$$

$$\gamma_{fi}^{+-} = \frac{2\pi}{\hbar}\sum_{j\geq k}\left(1 - \frac{1}{2}\delta_{jk}\right)\iint \left|\sum_c \frac{\langle f|\hat{V}_j|c\rangle\,\langle c|\hat{V}_k|i\rangle}{E_c - E_i + \hbar\omega_k} + \frac{\langle f|\hat{V}_k|c\rangle\,\langle c|\hat{V}_j|i\rangle}{E_c - E_i - \hbar\omega_j}\right|^2$$

$$\frac{\bar{n}_j}{2}\frac{(\bar{n}_k+1)}{2}\ \delta(E_f - E_i - \hbar\omega_j + \hbar\omega_k)\ \rho_j(\hbar\omega_j)\rho_k(\hbar\omega_k)\,\mathrm{d}\hbar\omega_j\,\mathrm{d}\hbar\omega_k \tag{5}$$

$$\gamma_{fi}^{-+} = \frac{2\pi}{\hbar}\sum_{j\geq k}\left(1-\frac{1}{2}\delta_{jk}\right)\iint\left|\sum_c \frac{\langle f|\hat{V}_j|c\rangle\,\langle c|\hat{V}_k|i\rangle}{E_c - E_i - \hbar\omega_k} + \frac{\langle f|\hat{V}_k|c\rangle\,\langle c|\hat{V}_j|i\rangle}{E_c - E_i + \hbar\omega_j}\right|^2$$

$$\frac{(\bar{n}_j+1)}{2}\frac{\bar{n}_k}{2}\ \delta(E_f - E_i + \hbar\omega_j - \hbar\omega_k)\ \rho_j(\hbar\omega_j)\rho_k(\hbar\omega_k)\,\mathrm{d}\hbar\omega_j\,\mathrm{d}\hbar\omega_k \tag{6}$$

Here, mode j has energy $\hbar\omega_j$ and Bose-Einstein occupation factor $\bar{n}_j = [\exp(\hbar\omega_j/k_{\mathrm{B}}T) - 1]^{-1}$. In our implementation of the (Raman) Orbach mechanisms, we consider the sum over all (pairs of) modes where each mode is considered to have an antilorentzian line shape (equation 7) with central energy μ_j and FWHM Γ_j. The minus and plus signs in the superscripts of γ_{fi} indicate the absorptions and emission, respectively, of the corresponding vibrational quanta by the spin system. In the calculation of Raman relaxation, we only consider transitions between the two states of the ground Kramers doublet and exclude vibrations with $\tilde{\nu} > 200\,\mathrm{cm}^{-1}$ indicated in figure S12. Restricting the integral to this low energy region prevents the break-down of the perturbative expressions in equations 3 to 6 by ensuring that the energy denominators do not approach zero, while still capturing the most important contributions to Raman relaxation. This also has the added benefit of enabling vast computational savings. To obtain the magnetic reversal rate, we diagonalise the rate matrix and take the smallest magnitude non-zero eigenvalue which corresponds to the negative rate of magnetic reversal.[4]

$$\rho_j(E) = \frac{\frac{\Gamma_j}{2\left(\left(\frac{\Gamma_j}{2}\right)^2+(E-\mu_j)^2\right)} - \frac{\Gamma_j}{2\left(\left(\frac{\Gamma_j}{2}\right)^2+(E+\mu_j)^2\right)}}{2\tan^{-1}\left(\frac{2\mu_j}{\Gamma_j}\right)} \tag{7}$$

S2 Displacement scans

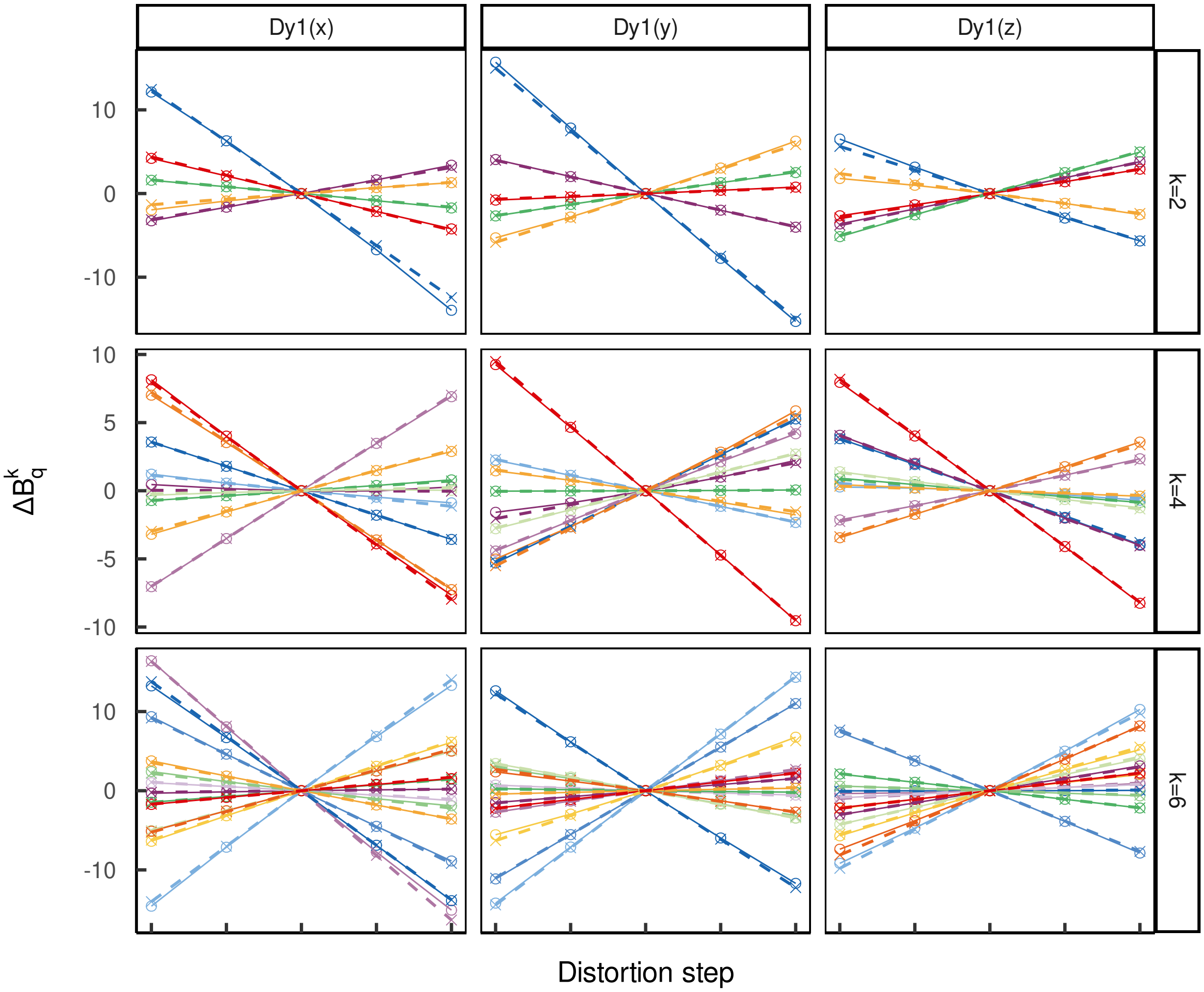

Figure S1: Scans of $\Delta B_q^k = B_q^k(\boldsymbol{r}) - B_q^k(0)$ along the atomic displacements of the dysprosium ion. Different colours indicate different orders q (green: $q = 0$, cold: $q < 0$, warm: $q > 0$). Values obtained by explicit finite displacement sampling are shown by solid lines and circles, and values predicted by the Linear Vibronic Coupling (LVC) method are shown by dashed lines and crosses.

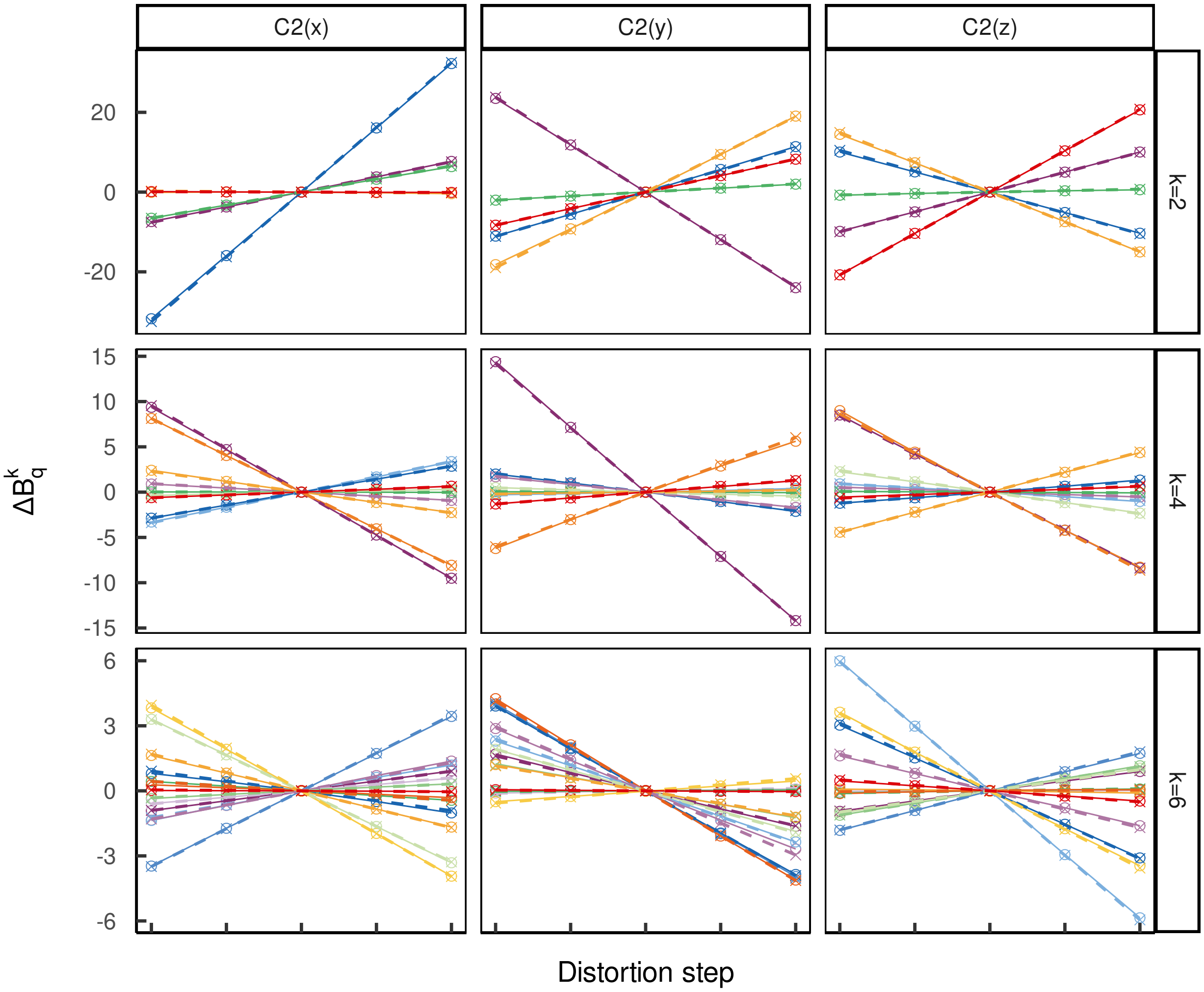

Figure S2: Scans of $\Delta B_q^k = B_q^k(\boldsymbol{r}) - B_q^k(0)$ along the atomic displacements of a cyclobutadienyl (Cb) ring carbon atom. Different colours indicate different orders q (green: $q = 0$, cold: $q < 0$, warm: $q > 0$). Values obtained by explicit finite displacement sampling are shown by solid lines and circles, and values predicted by the LVC method are shown by dashed lines and crosses.

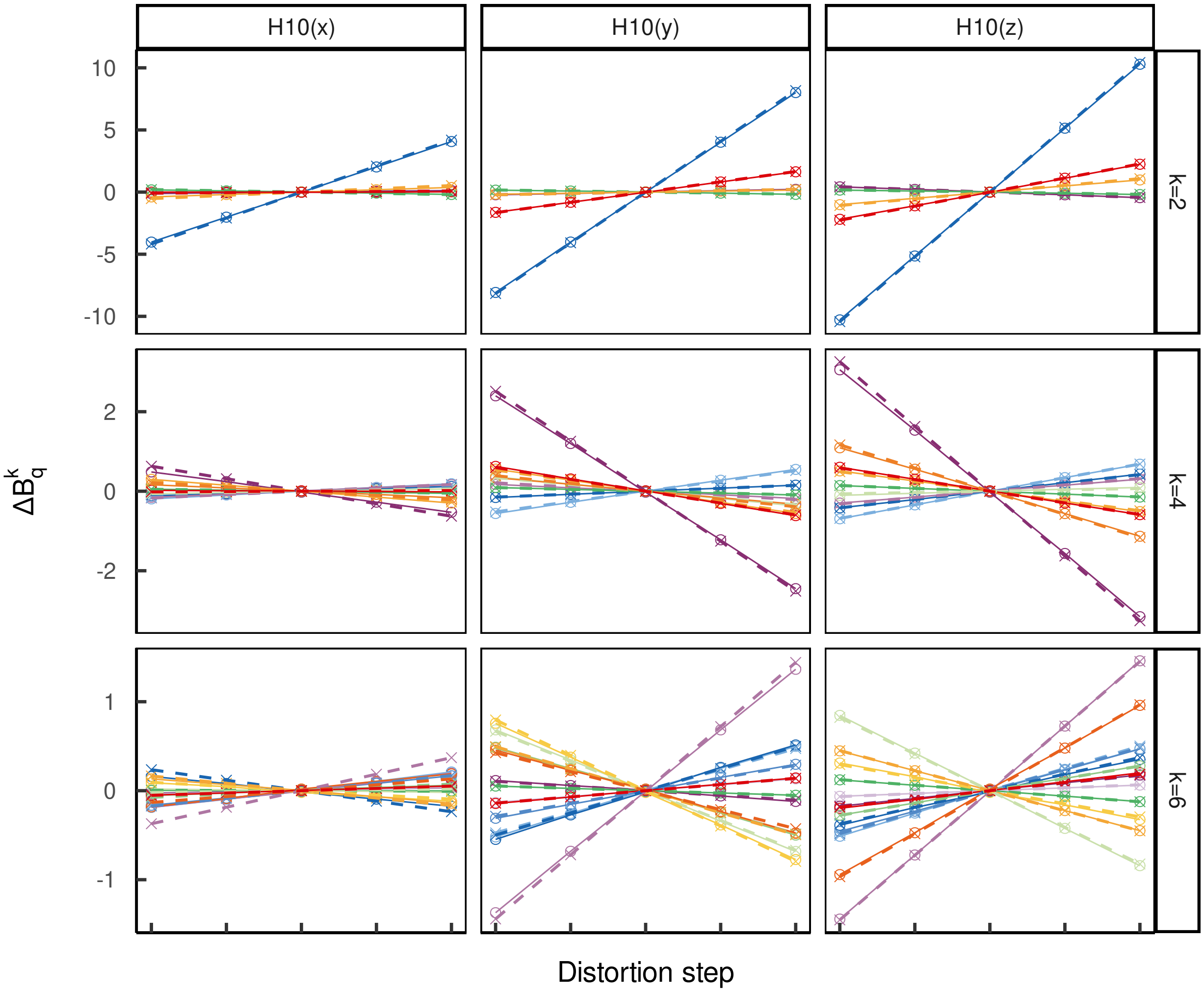

Figure S3: Scans of $\Delta B_q^k = B_q^k(\boldsymbol{r}) - B_q^k(0)$ along the atomic displacements of a Cb hydrogen atom. Different colours indicate different orders q (green: $q = 0$, cold: $q < 0$, warm: $q > 0$). Values obtained by explicit finite displacement sampling are shown by solid lines and circles, and values predicted by the LVC method are shown by dashed lines and crosses.

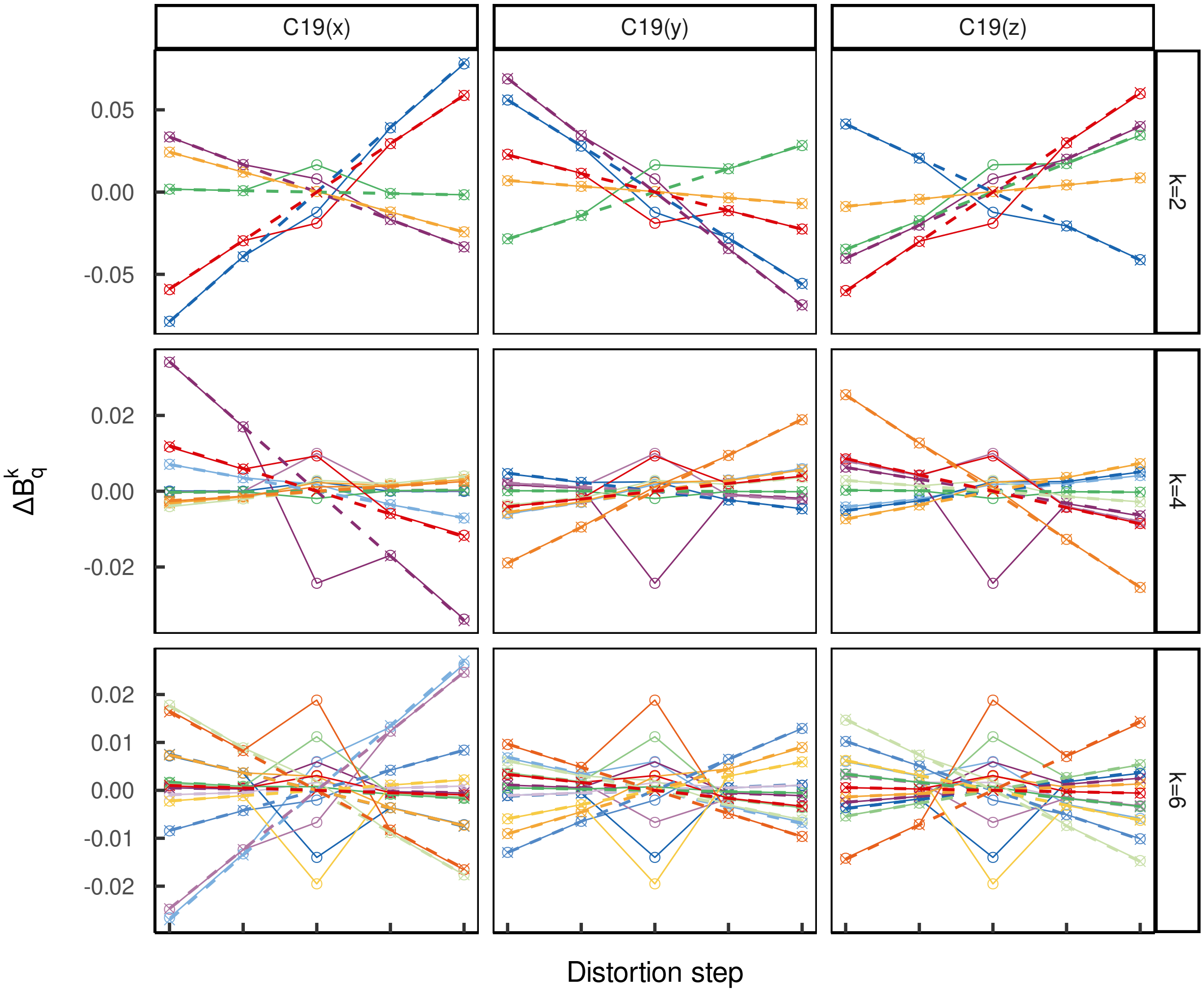

Figure S4: Scans of $\Delta B_q^k = B_q^k(\boldsymbol{r}) - B_q^k(0)$ along the atomic displacements of an environmental dichloromethane (DCM) carbon atom. Different colours indicate different orders q (green: $q = 0$, cold: $q < 0$, warm: $q > 0$). Values obtained by explicit finite displacement sampling are shown by solid lines and circles, and values predicted by the LVC method are shown by dashed lines and crosses.

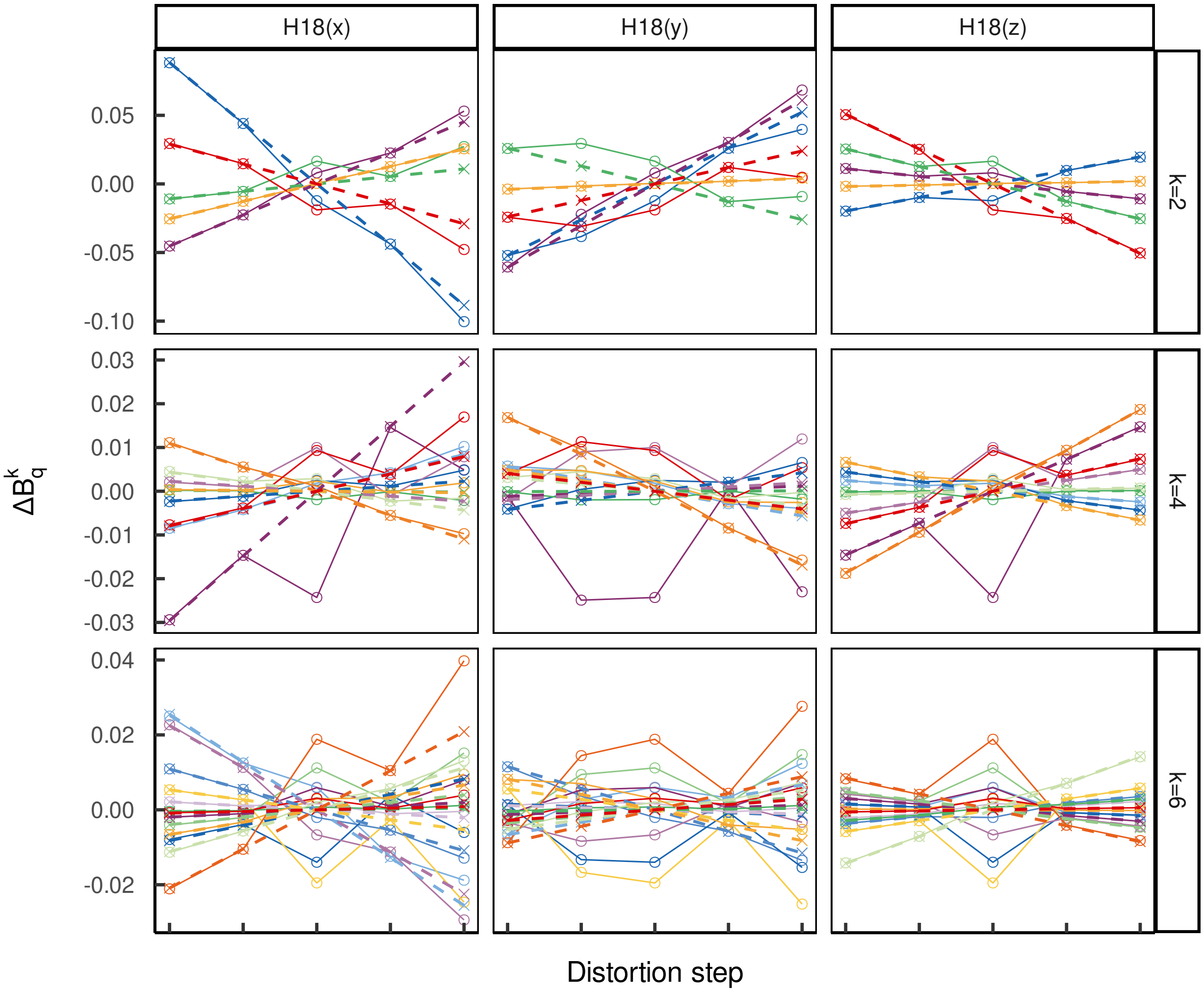

Figure S5: Scans of $\Delta B_q^k = B_q^k(\boldsymbol{r}) - B_q^k(0)$ along the atomic displacements of an environmental DCM hydrogen atom. Different colours indicate different orders q (green: $q = 0$, cold: $q < 0$, warm: $q > 0$). Values obtained by explicit finite displacement sampling are shown by solid lines and circles, and values predicted by the LVC method are shown by dashed lines and crosses.

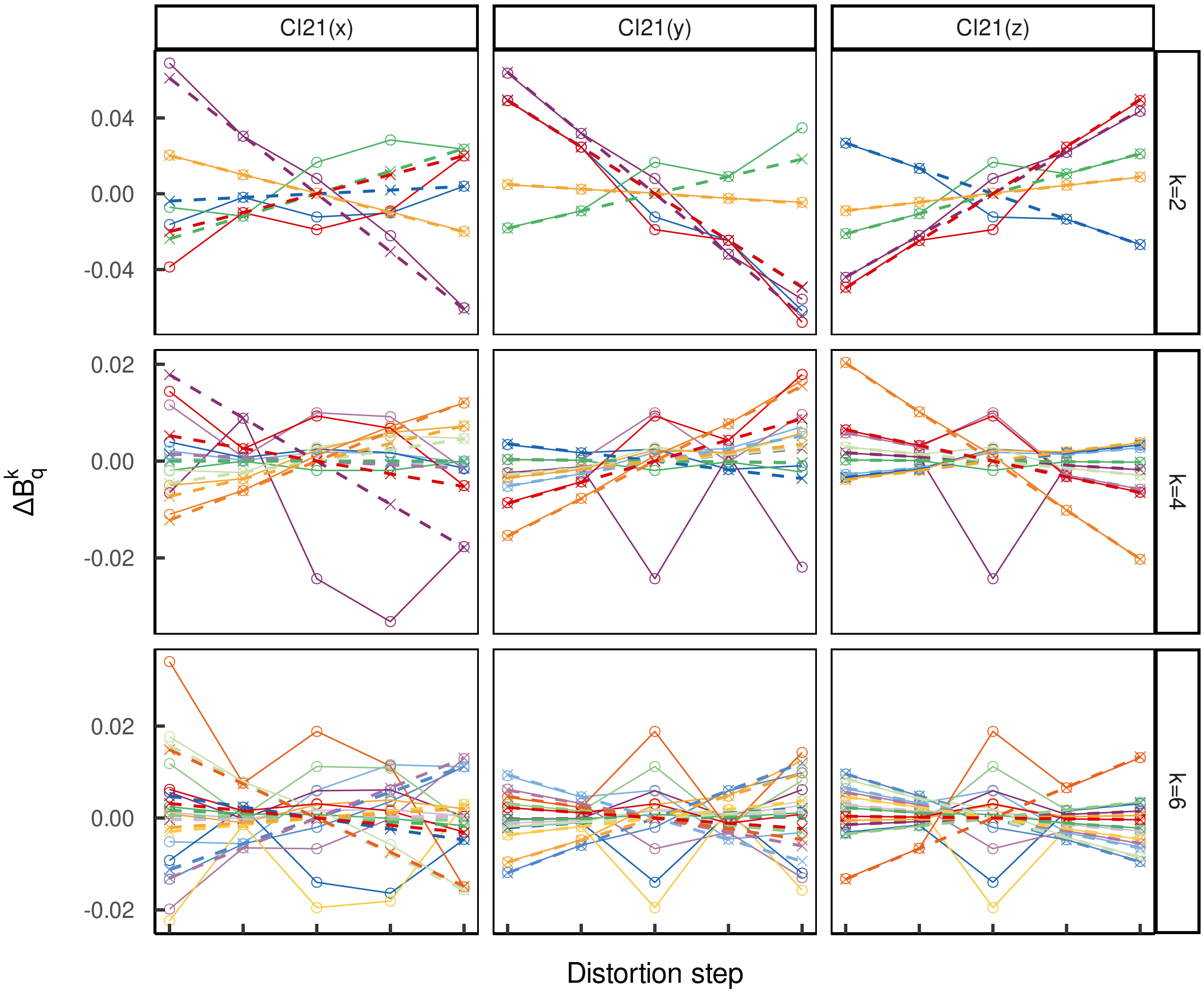

Figure S6: Scans of $\Delta B_q^k = B_q^k(\boldsymbol{r}) - B_q^k(0)$ along the atomic displacements of an environmental DCM chlorine atom. Different colours indicate different orders q (green: $q = 0$, cold: $q < 0$, warm: $q > 0$). Values obtained by explicit finite displacement sampling are shown by solid lines and circles, and values predicted by the LVC method are shown by dashed lines and crosses.

S3 Relative deviation of the differentiation methods

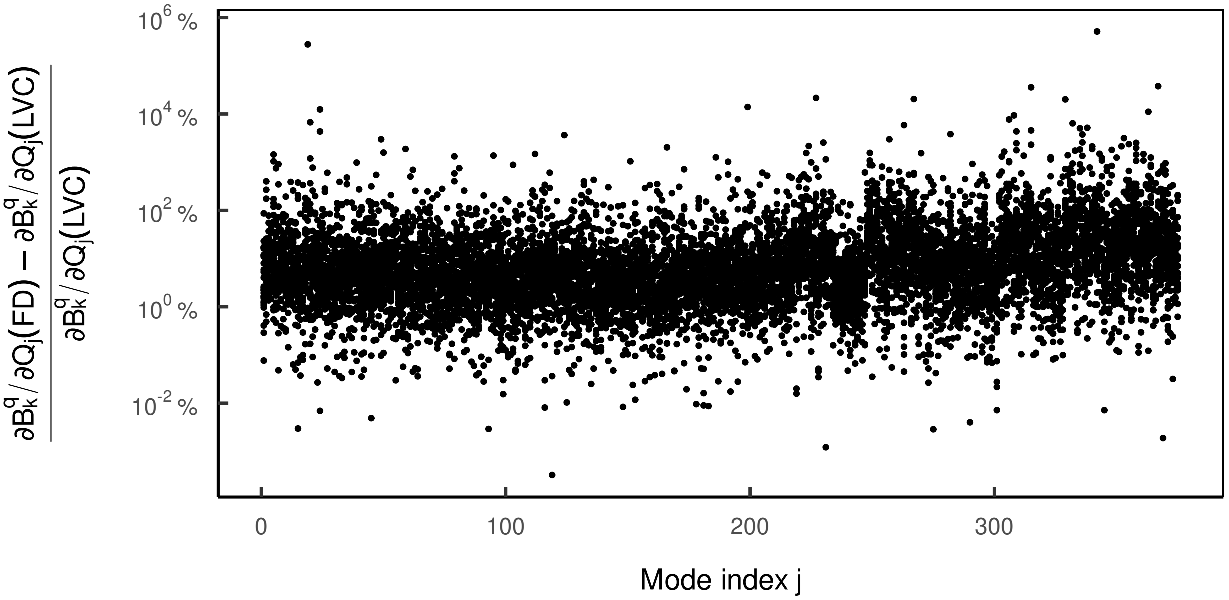

Figure S7: Relative deviation of the Crystal Field Parameters (CFPs) derivatives in the normal mode basis. Large relative errors are a result of the sever effect of numerical noise in the case of small magnitude derivatives. The typical relative deviation is observed to be 1 %–100 %.

S4 Translational invariance of numerical and analytic derivatives

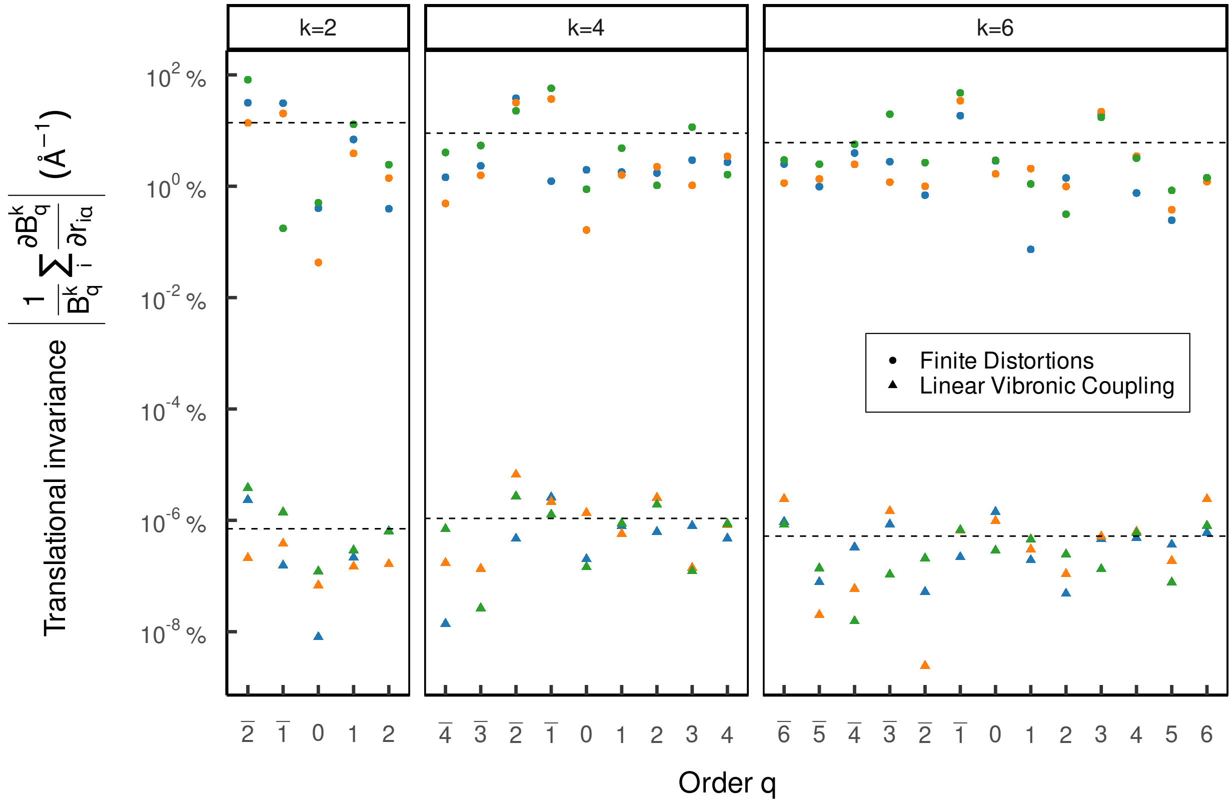

Figure S8: Violation of the translational invariance condition (equation 22 in the main text) of the B_q^k parameter derivatives by rank k and order q per Å translation along each Cartesian axis. Large values reflect strong violation of the translational invariance condition. Different colours indicate different Cartesian directions $\alpha \in \{x, y, z\}$.

S5 Computational benchmark of derivation method

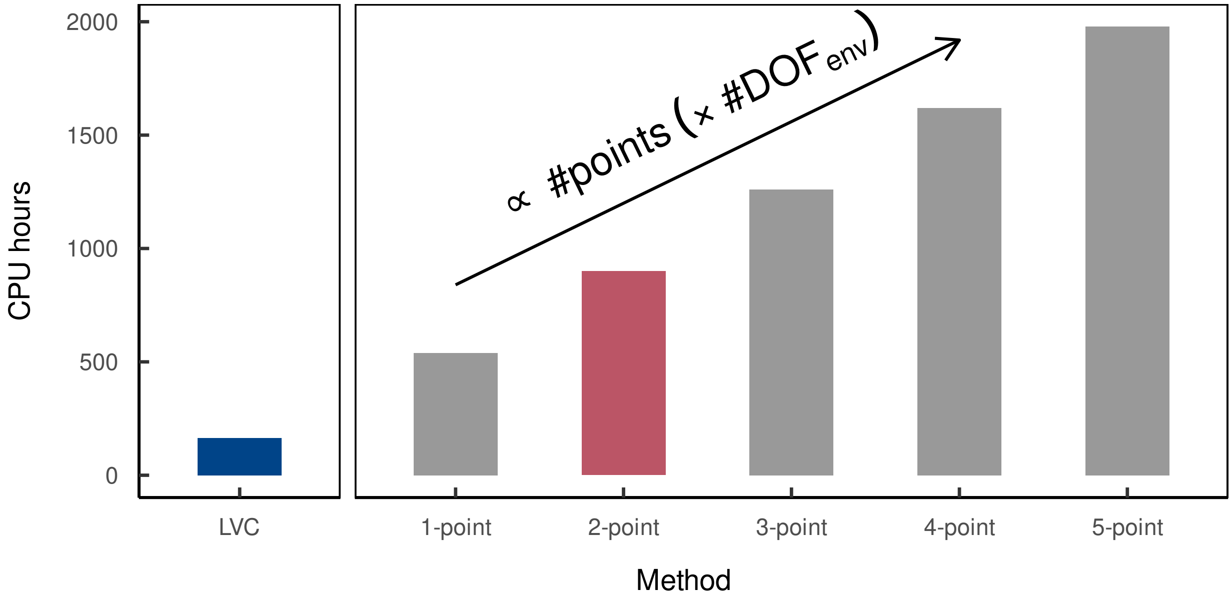

Figure S9: Computational benchmark of the LVC derivatives versus n-point central finite difference methods. While the evaluation of finite difference derivative scales linearly with the number of steps n and number of environment Degrees of Freedom (DOFs), the computational cost of LVC derivatives is independent of the latter.

S6 Full correlation matrix of CFP derivatives

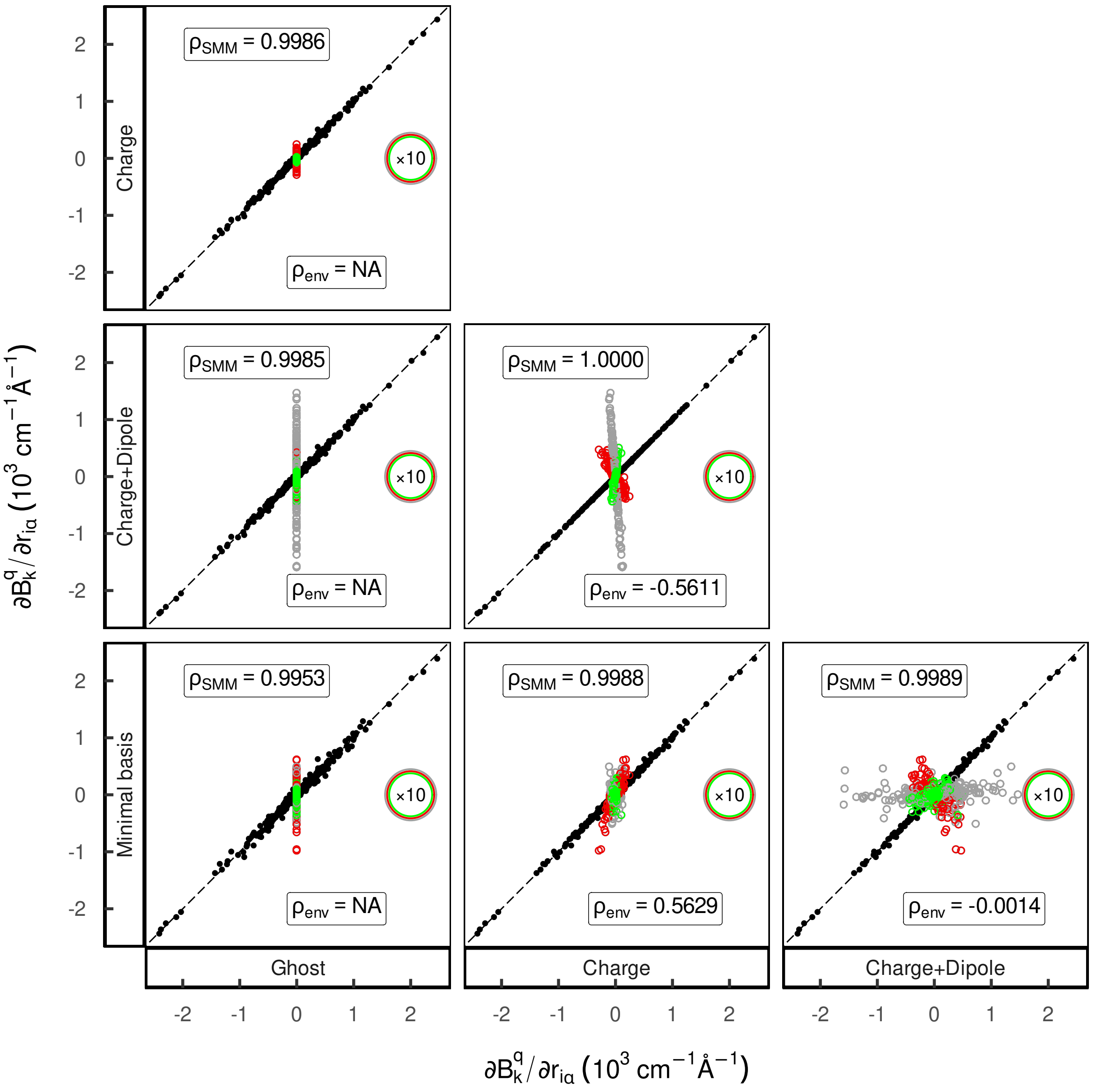

Figure S10: Mutual correlation scatter plots of the atomic CFP derivatives between different environment representations. Environmental and Single-Molecule Magnet (SMM) DOFs are depicted with unfilled and solid dots, respectively. The values of CFP derivatives associated to environmental DOFs are scaled by a factor of 10 for better visibility.

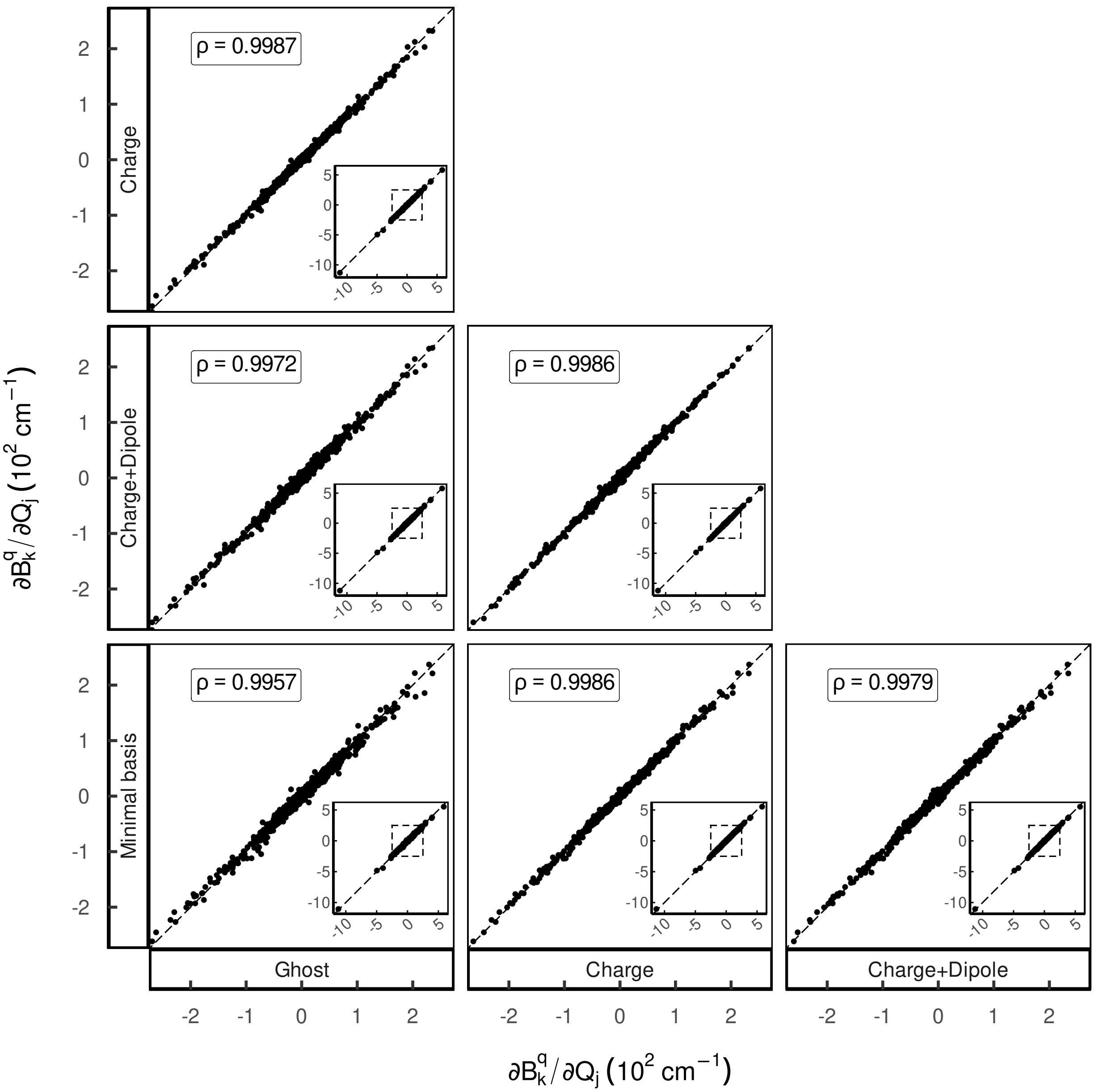

Figure S11: Mutual correlation scatter plots of the CFP derivatives in the normal mode basis between different environment representations.

Here, we further elaborate on role the of the charge magnitude and sign in the poor agreement of atomic CFP derivatives along the environmental DOFs observed at the charge+dipole

approximation mentioned in section 4.2 of the main text. The anticorrelation in the carbon and hydrogen DOFs data observed in the charge-only versus charge+dipole correlation plots, shown in figure S10, reflects the change in sign between charge-only and charge+dipole model, and the significant increase in charge magnitude leads to overall stronger coupling. The charge of the chlorine atoms remains unchanged in sign but increases in magnitude upon introduction of dipoles. Again, this leads to a stronger coupling of equal sign in the charge+dipole model of the environment as the electrostatic monopole terms appears to dominate the modulation of the crystal field.

S7 Vibrational density of states

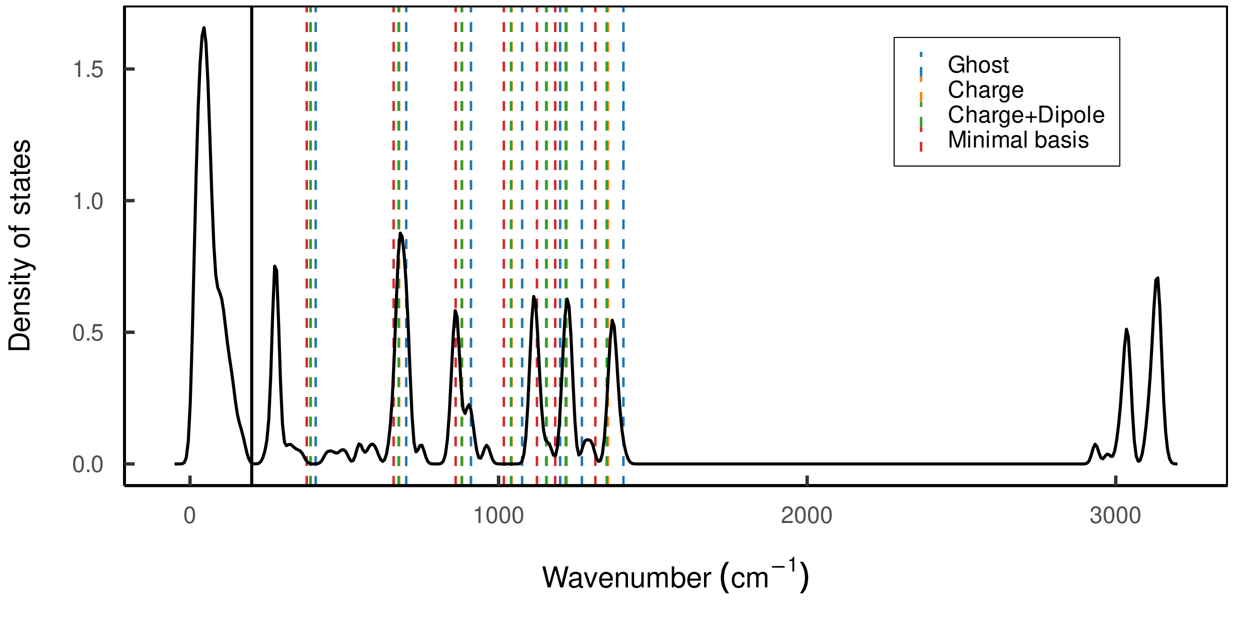

Figure S12: Vibrational Density Of States (DOS) with the position of the electronic states indicated by dashed lines. The solid black line at 200 cm^{-1} indicates the cutoff used for the calculation of Raman rates.

References

(1) Reta, D.; Kragskow, J. G. C.; Chilton, N. F. Ab Initio Prediction of High-Temperature Magnetic Relaxation Rates in Single-Molecule Magnets. *Journal of the American Chemical Society* **2021**, *143*, 5943–5950, DOI: 10.1021/jacs.1c01410.

(2) Goodwin, C. A. P.; Ortu, F.; Reta, D.; Chilton, N. F.; Mills, D. P. Molecular magnetic hysteresis at 60 kelvin in dysprosocenium. *Nature* **2017**, *548*, 439–442, DOI: 10.1038/nature23447.

(3) Lunghi, A. Toward exact predictions of spin-phonon relaxation times: An ab initio implementation of open quantum systems theory. *Science Advances* **2022**, *8*, DOI: 10.1126/sciadv.abn7880.

(4) Alexander, M. H.; Hall, G. E.; Dagdigian, P. J. The Approach to Equilibrium: Detailed Balance and the Master Equation. *Journal of Chemical Education* **2011**, *88*, 1538–1543, DOI: 10.1021/ed2001329.
